# SU(*N*) symmetry of coherent photons controlled by rotated waveplates

**DOI:** 10.1016/j.heliyon.2024.e34423

**Published:** 2024-07-14

**Authors:** Shinichi Saito

**Affiliations:** Center for Exploratory Research Laboratory, Research & Development Group, Hitachi, Ltd., 1-280 Higashi-Koigakubo, Kokubunji, 185-8601, Tokyo, Japan

**Keywords:** Lie algebra, SU(*N*), Gell-Mann sphere, Orbital angular momentum, Spin angular momentum, Coherent state

## Abstract

The coherent state from a laser source has spin and orbital degrees of freedom, which allow an arbitrary superposition state among orthogonal states with varying amplitudes and phases. Here, we theoretically show coherent photons with SU(*N*) symmetry are characterised by expectation values of angular momentum shown on a hypersphere in SO($N^{2}-1$) space. To demonstrate expected unitary transformations in experiments, we have constructed generators of transformations in the Lie group simply by combining widely available optical components such as waveplates and vortex lenses. We show a superposition state between twisted and Gaussian states is characterised by the dynamics of the topological charge upon the transformation in SU(3) states. We also realised photonic singlet and triplet states corresponding to SU(4) states, which were projected to SU(2)×SU(2) states upon passing through a rotated polariser.

## 1 Introduction

Structured lights are attracting significant attentions for more than three decades [1], [2], [3], [4], [5], [6], [7], [8], [9], [10], [11], [12], [13], [14], [15], [16], [17], [18], [19], [20], [21], [22], and yet, a lot of new discoveries are still being reported, which include realisation of classical and quantum entanglement [23], [24], [25], [26], [27], [28], [14], [15], [29], [30], [31], [32], Greenberger–Horne–Zeilinger (GHZ) states [20], and optical skyrmions [33], [34] to name a few. The noble properties are coming from non-separability of structured lights [23], [35], [30], [17], [36], [18], [19], [20], [32], [37], [10], [16], [38], [39], [31], [21], [22] with fundamental degrees of freedom such as spin and orbital angular momentum [1], [2], [3], [4], [5], [6], [7], [8], [9], [10], [11], [12], [13], [14], [15], [16], [17], [18], [19], [20], [21], [22]. The classical entanglement is limited to be the local correlation [23], such that it will not induce non-local correlation as inspired by the Einstein-Podolsky-Rosen (EPR) paradox [40], [24], [25]. Nevertheless, the classical entanglement is expected to be promising for new innovations in sensing, metrology, communications, and computation [30], [32], [16], [37]. As proposed by Spreeuw [23], the classical entanglements can be easily visualised as far-field images [23], [26], [27], [28], [14], [15], [37], [29], [30], [38], [39], compared with the more sophisticated experiments in single photons [41], [42], [43], [44], [45], [46].

One of the reasons why we are attracted by structured lights is coming from their topological features [21], [22], [34] inherent to the optical vortices [47], [7], [31], [32]. It is particularly interesting to recognise the internal structure of coherent states, as shown on the higher order Poincaré spheres [1], [2], [3], [4], [5], [6], [7], [8], [9], [10], [11], [12], [13], [14], [15], [16], [17], [18], [19], [20], [21], [22], [48]. The Poincaré spheres correspond to the macroscopic coherence of internal quantum states similar to the Bloch sphere for spin of a single electron [49]. The notion of macroscopic quantum phenomena is applicable only to the limited number of systems, such as superfluid He [50], [51], [52], [53], superconductors [54], [55], [56], [57], cold atoms [58], [59], and lasers [60], [61], [62], [63], [64]. These systems would be useful as platforms to explore why our macroscopic classical world seems to be different from the microscopic world, governed by quantum mechanics [65], [66], [67], [68].

These classical examples of macroscopic quantum phenomena might be less exotic, however, compared with Schrödinger's cat state [65], [66], [67], [68], since macroscopic number of elementary particles simply occupy the same state, due to the nature of Bose-Einstein Condensation (BEC) upon broken symmetry [69], [70], [71], [72], [73], [74], [75], [76]. Nevertheless, it is still intriguing to treat the whole system by the sole wavefunction. This is easily understood for bosons [58], [59], [72], [73], [74], [75], [76], since quantum statistics allows bosons to occupy the same energy state, such that the macroscopic number of bosons simply occupy the same state if the interaction is weak to expect BEC at low enough temperatures. Even for degenerate fermions, such as electrons in a metal with weak attractive interaction, Cooper pairs are formed between time-reversal symmetric spin-up and down electrons, which behave like bosons [57] leading to a spontaneous U(1) (the unitary group of one-dimension) symmetry breaking to exhibit superconducting behaviour below a critical temperature [54], [77], [55], [56], [57] similar to BEC [52], [53]. Consequently, the system is described by the macroscopic wavefunction of the Ginzburg-Landau equation [56], [72], [73]. After the broken U(1) symmetry, there exist collective modes, named Nambu-Anderson-Higgs-Goldstone modes [69], [70], [71], [77], whose energy must vanish in the long wavelength limit to recover the symmetry of the system before the phase-transition. In superconductors, these collective modes are difficult to observe, since electrons have charge, such that the phase fluctuation induces plasma oscillations [56], [72], [73], while similar collective modes were observed in cold atoms as Bogoliubov modes [58], [59]. In addition to these quasi-particle excitations, the beauty of BEC lies in the fact, that the entire system is described by the U(1) wavefunction of the Gross-Pitaevskii equation [58], [59]. Consequently, the whole system behaves quantum mechanically to exhibit interferences as matter waves [58], [59].

We have recently revisited [78], [79], [80], [81], [82], [83], [84], [85], [48], [86] for the notion of the macroscopic coherence in the case of photons emitted from a laser source [87], [88], [89], [90], [91], [92], [93], [94], [60], [61], [62], [63], [64]. Our hypothesis is that we can describe the coherent state of photons as a macroscopic wavefunction to exhibit quantum mechanical behaviours at least for spin and orbital angular momentum states. We believe this hypothesis is highly non-trivial, since the state above lasing threshold is often considered to be classical, following to Maxwell equations [93], [94], [60], [61], [62], [63], [64] rather than photonic Dirac equations [95], [96], [97], [98], [99], [100], [101], [102], [103], [81]. However, do we really have classical photons? Photons are elementary particles, such that they are different from classical macroscopic objects and they must be described by a wavefunction. Then, in what sense can we consider the coherent state as classical electromagnetic waves [93], [94]? In particular, the calculus of Jones vectors for Stokes parameters $\left(S_{1},S_{2},S_{3}\right)$ on the Poincaré sphere is highly quantum mechanical, which is completely consistent with the two-level systems for spin [87], [88], [89], [90], [91], [92], [93], [94], [60], [61], [62], [63], [64], [78] even if macroscopic number of photons are involved. Why classical electromagnetic waves behave quantum mechanically for a polarisation state? And, perhaps more importantly, what makes the apparent quantum-mechanical behaviour to be classical ceasing the coherence?

We have addressed some of these questions [78], [79], [80], [81], [82], [83], [84], [85], [48], and so far, it is consistent with the view that coherent states for spin angular momentum can be described by macroscopic wavefunctions with the SU(2) (the special unitary group of degree two) symmetry. Above lasing threshold for pumping [60], [61], [62], [63], [64], the SU(2) symmetry of photons related to the rotational symmetry upon the propagation is broken [81] due to the *natural selection* of the mode with the lowest loss for stimulated emissions in a laser cavity. For coherent photons from a laser source, there is no energy required to rotate the polarisation state to recover the SU(2) symmetry [78], [81], since photons do not have charge nor the magnetic moment. There is no interaction among photons in a vacuum, and only weak non-linear interaction is taking place in certain families of materials [60], [61], [62], [63], [64]. Consequently, the SU(2) wavefunction describes the polarisation state, which is characterised by the spin expectation values shown on Poincaré sphere as Stokes parameters [87], [88], [89], [90], [91], [92], [93], [94], [60], [61], [62], [104], [105], [106], [107], [108], [63], [64], [23], [49], [109], [19], [78]. We have also examined orbital angular momentum [1], [2], [3], [4], [5], [7], [8], [9], [10], [11], [12], and found that the orbital angular momentum is a proper quantum-mechanical observable with SU(2) symmetry in a waveguide or in a free space if the mode is predominantly propagating along a paraxial direction with a sufficient collimation [79], [80], [48]. If we combine left and right vortices and a no-twisted state, we theoretically expected to realise an SU(3) state, similar to colour charge for quarks in Quantum Chromo-Dynamics (QCD) [110], [111], [112], [113], [114], [115], [116]. A representation theory of the Lie group [117], [118], [119], [113], [96] was employed to calculate expectation values for generators of transformations in the su(3) Lie algebra, which form a hypersphere of SO(8) (the special orthogonal group of degree eight) symmetry named as a Gell-Mann hypersphere [48]. The SU(3) state can also be characterised by two Poincaré spheres; one for orbital angular momentum and the other for hyperspin [48] to represent couplings between a twisted state and a no-twisted state.

The purpose of this paper is to extend the theory to analyse coherent photons with SU(*N*) symmetry of arbitrary integer degree *N* and to examine the proposed states by free-space optical experiments. We explore how topological charge is responsible for changing from a twisted state to a no-twisted state under a fixed polarisation state for SU(3) states. Moreover, we also included spin degree of freedom together with orbital angular momentum to realise photonic singlet and triplet states as SU(4) states. We provide simple methods to manipulate both amplitudes and phases for SU(3) and SU(4) states by using rotated waveplates.

## 2 Theory

Here, we describe a theory to realise various superposition states of coherent photons with spin and orbital angular momentum [23], [26], [27], [28], [15], [29], [30][19][120], [121], [122], [123] [78][80][83][85][48]. We show that the coherency is preserved upon the SU(*N*) transformation and establish the relationship between the SU(*N*) state and the expectation values of the angular momentum.

### 2.1 Coherent photons with spin and orbital angular momentum

Historically, it was considered in the past that spin and orbital angular momentum cannot be split into the independent observables for photons in a gauge invariant way [124], [125], [126], [127]. However, it was recently shown that it is possible to define spin and orbital angular momentum operators with SO(3) symmetry [128] or SU(2) symmetry [80] for photons propagating in a waveguide or free space under sufficient collimation [80]. If we accept the orthogonality condition, the modes with different orbital angular momentum of *m* or different radial node of *n* are orthogonal to each other [2][79], even if spin angular momentum of *σ* is the same. Similarly, if states have orthogonal spin, they are orthogonal to each other, even if orbital angular momentum is the same. Then, the orthonormality is simply described as $\langle \sigma ,m,n|\sigma^{\prime },m^{\prime },n^{\prime }\rangle =\delta_{\sigma ,\sigma^{\prime }}\delta_{m,m^{\prime }}\delta_{n,n^{\prime }}$, where *δ* is the Kronecker delta. We can label the quantum state by an integer (i∈Z), such that we assign *i* for (σ,m,n). If one of the quantum numbers of σ,m,n are different, we assign different value of *i*. If there are *N* orthogonal states (i=1⋯N), the arbitrary superposition state among these states are described by SU(*N*) states [110][111][112][113][114][115][116][48]. There are pioneering works [120], [121], [122], [123] in photonics to explore and utilise the SU(*N*) symmetry in arrays of multi-port interferometers [121], [122], [123]. First, we will examine SU(2) states among Left (L) and Right (R) states for orbital angular momentum [2][3][4][5] in our experiments while polarisation state is the same. We will also explore SU(3) states among LR states and a no-twisted state (O) at fixed polarisation [48], and examine SU(4) states with variable mixtures of L and R for orbital angular momentum under horizontal (H) and vertical (V) polarisation states.

So far, we have discussed only for a single-particle state. Next, we will consider a quantum field theory to describe many-body states for coherent photons. We define creation and annihilation operators for a photon for the state of *i* as ${\overset{ˆ}{a}}_{i}^{†}$ and ${\overset{ˆ}{a}}_{i}$, respectively. These operators must satisfy the Bose-Einstein commutation relationships of $[{\overset{ˆ}{a}}_{i},{\overset{ˆ}{a}}_{j}^{†}]=\delta_{i,j}$, $[{\overset{ˆ}{a}}_{i}^{†},{\overset{ˆ}{a}}_{j}^{†}]=0$, and $[{\overset{ˆ}{a}}_{i},{\overset{ˆ}{a}}_{j}]=0$. Then, we can describe a single-particle state by the creation operator as $|i\rangle ={\overset{ˆ}{a}}_{i}^{†}|0\rangle$, which is equivalent to $|\sigma ,m,n\rangle ={\overset{ˆ}{a}}_{\sigma ,m,n}^{†}|0\rangle$, where |0〉 is the vacuum state.

The coherent state with the SU(*N*) symmetry is given by the many-body state,

(1) $$|\alpha_{1},\cdots ,\alpha_{N}\rangle =\prod_{i=1}^{N}e^{-\frac{|\alpha_{i}{|}^{2}}{2}}e^{{\overset{ˆ}{a}}_{i}^{†}\alpha_{i}}|0\rangle ,$$

where $|I\rangle =\alpha ={\left(\alpha_{1},\cdots ,\alpha_{N}\right)}^{t}$ is the SU(*N*) state for the initial state, given by *N*-dimensional complex vector of $C^{N}$ and ^t^ stands for the transpose operation. As we shall see below, ***α*** works as a macroscopic wavefunction of coherent photons. In order to execute an arbitrary operation to transfer |I〉 to the final state of $|F\rangle =\alpha^{\prime }={\left(\alpha_{1}^{\prime },\cdots ,\alpha_{N}^{\prime }\right)}^{t}$, we need $N^{2}-1$ generators of transformations in su(N) Lie algebra, $A_{i}$, which are made of N×N matrices [110][111][112][113][114][115][116][118][119][48]. $A_{i}$ satisfies the commutation relationship of $[A_{i},A_{j}]=i\sum_{k}f_{ijk}A_{k}$, where $i,j,k=1\cdots (N^{2}-1)$ and $f_{ijk}$ is the structure constant [115][113][118][119][48]. The generators of unitary transformations are physically linked to generalised angular momentum operators with the component of $i=1,\cdots (N^{2}-1)$ in the many-body Nambu representation [71][56], defined as ${\overset{ˆ}{A}}_{i}=\hbar {\overset{ˆ}{\psi }}^{†}A_{i}\overset{ˆ}{\psi }$, where ${\overset{ˆ}{\psi }}^{†}=({\overset{ˆ}{a}}_{1}^{†},\cdots ,{\overset{ˆ}{a}}_{N}^{†})$ and $\overset{ˆ}{\psi }={\left({\overset{ˆ}{a}}_{1},\cdots ,{\overset{ˆ}{a}}_{N}\right)}^{t}$ are field operators for creations and annihilations of photons. Then, the rotational operator is given by the exponential map,

(2) $${\overset{ˆ}{D}}_{i}(\delta \phi )=\exp\left(-\frac{i}{\hbar }{\overset{ˆ}{A}}_{i}\frac{\delta \phi }{2}\right),$$

where the amount of rotation is determined by δϕ/2. Then, it is straightforward to calculate the impact of the rotation as

(3) $$|\alpha_{1}^{\prime },\cdots ,\alpha_{N}^{\prime }\rangle ={\overset{ˆ}{D}}_{i}(\delta \phi )\prod_{i=1}^{N}e^{-\frac{|\alpha_{i}{|}^{2}}{2}}e^{{\overset{ˆ}{a}}_{i}^{†}\alpha_{i}}|0\rangle$$

(4) $$=\prod_{i=1}^{N}e^{-\frac{|\alpha_{i}^{\prime }{|}^{2}}{2}}e^{{\overset{ˆ}{a}}_{i}^{†}\alpha_{i}^{\prime }}|0\rangle ,$$

where we obtain the transferred SU(*N*) wavefunction

(5) $$\alpha^{\prime }=D_{i}(\delta \phi )\alpha ,$$

where

(6) $$D_{i}(\delta \phi )=\exp\left(-iA_{i}\frac{\delta \phi }{2}\right).$$

This means that the coherent state is kept its form to maintain the coherence upon the SU(*N*) rotation [78][79][80][48]. This is coming from the nature of coherent photons, similar to the BEC, where macroscopic number of bosons are condensed to the lowest energy level [66] [53][59][50][52][57][68][129]. Therefore, we can expect the SU(*N*) transformation is applicable to the coherent photons.

### 2.2 Proof of the coherency after the SU(*N*) transformation

The coherent state of Equation (1) is the exponential map of the creation operator [130][114][115][116][78][48], while the SU(*N*) state of ***α*** is transferred by the exponential map of the su(N) Lie algebra [115][113][118][119][78][48] shown in Equation (5). Consequently, Equation (4) is given by *the exponential map of the exponential map.* We think this is not trivial and it is worth for showing the mathematical proof of Equation (4) for the first time.

To prove the formula, it is useful to obtain the commutation relationship between ${\overset{ˆ}{D}}_{i}(\delta \phi )$ and ${\overset{ˆ}{a}}_{j}^{†}$. To achieve it we will calculate ${\overset{ˆ}{D}}_{i}(\delta \phi ){\overset{ˆ}{a}}_{j}^{†}{\overset{ˆ}{D}}_{i}^{-1}(\delta \phi )$, using the Campbell-Baker-Hausdorff formula [91][92]

(7) $$e^{\overset{ˆ}{X}}\overset{ˆ}{Y}e^{-\overset{ˆ}{X}}=\overset{ˆ}{Y}+[\overset{ˆ}{X},\overset{ˆ}{Y}]+\frac{1}{2}[\overset{ˆ}{X},[\overset{ˆ}{X},\overset{ˆ}{Y}]]+\frac{1}{3!}[\overset{ˆ}{X},[\overset{ˆ}{X},[\overset{ˆ}{X},\overset{ˆ}{Y}]]]+\cdots ,$$

where we should put

(8) $$\overset{ˆ}{X}=-\frac{i}{\hbar }{\overset{ˆ}{A}}_{i}\frac{\delta \phi }{2}$$

(9) $$=-i\sum_{k,l}{\overset{ˆ}{a}}_{k}^{†}{\left(A_{i}\right)}_{kl}{\overset{ˆ}{a}}_{l}\frac{\delta \phi }{2},$$

and $\overset{ˆ}{Y}={\overset{ˆ}{a}}_{j}^{†}$. We successively obtain

(10) $$[\overset{ˆ}{X},\overset{ˆ}{Y}]=(-i)\sum_{k}{\overset{ˆ}{a}}_{k}^{†}{\left(A_{i}\right)}_{kj}\left(\frac{\delta \phi }{2}\right)$$

(11) $$[\overset{ˆ}{X},[\overset{ˆ}{X},\overset{ˆ}{Y}]]={\left(-i\right)}^{2}\sum_{k,k^{\prime }}{\overset{ˆ}{a}}_{k}^{†}{\left(A_{i}\right)}_{kk^{\prime }}{\left(A_{i}\right)}_{k^{\prime }j}{\left(\frac{\delta \phi }{2}\right)}^{2}$$

(12) $$[\overset{ˆ}{X},[\overset{ˆ}{X},[\overset{ˆ}{X},\overset{ˆ}{Y}]]]={\left(-i\right)}^{3}\sum_{k,k^{\prime },k^{\prime \prime }}{\overset{ˆ}{a}}_{k}^{†}{\left(A_{i}\right)}_{kk^{\prime }}{\left(A_{i}\right)}_{k^{\prime }k^{\prime \prime }}{\left(A_{i}\right)}_{k^{\prime \prime }j}{\left(\frac{\delta \phi }{2}\right)}^{3},$$

and so on. Therefore, we obtain

(13) $$e^{\overset{ˆ}{X}}\overset{ˆ}{Y}e^{-\overset{ˆ}{X}}=\sum_{k}{\overset{ˆ}{a}}_{k}^{†}[\delta_{kj}+(-i){\left(A_{i}\right)}_{kj}\left(\frac{\delta \phi }{2}\right)+\frac{1}{2}{\left(-i\right)}^{2}\sum_{k^{\prime }}{\left(A_{i}\right)}_{kk^{\prime }}{\left(A_{i}\right)}_{k^{\prime }j}{\left(\frac{\delta \phi }{2}\right)}^{2}\;\;+\frac{1}{3!}{\left(-i\right)}^{3}\sum_{k^{\prime },k^{\prime \prime }}{\left(A_{i}\right)}_{kk^{\prime }}{\left(A_{i}\right)}_{k^{\prime }k^{\prime \prime }}{\left(A_{i}\right)}_{k^{\prime \prime }j}{\left(\frac{\delta \phi }{2}\right)}^{3}+\cdots ]$$

(14) $$=\sum_{k}{\overset{ˆ}{a}}_{k}^{†}{\left(e^{-iA_{i}\frac{\delta \phi }{2}}\right)}_{kj},$$

which gives a useful formula

(15) $${\overset{ˆ}{D}}_{i}(\delta \phi ){\overset{ˆ}{a}}_{j}^{†}=\sum_{k}{\overset{ˆ}{a}}_{k}^{†}{\left(D_{i}(\delta \phi )\right)}_{kj}{\overset{ˆ}{D}}_{i}(\delta \phi ).$$

The commutation relationship becomes

(16) $$[{\overset{ˆ}{D}}_{i}(\delta \phi ),{\overset{ˆ}{a}}_{j}^{†}]=\sum_{k}{\overset{ˆ}{a}}_{k}^{†}{\left(D_{i}(\delta \phi )-1\right)}_{kj}{\overset{ˆ}{D}}_{i}(\delta \phi ),$$

where **1** is the identity matrix in *N* dimensions. Note ${\overset{ˆ}{D}}_{i}(\delta \phi )$ contains creation and annihilation operators for many-body states, while $D_{i}(\delta \phi )$ is free from these operators for single-particle states. This means whenever we exchange ${\overset{ˆ}{D}}_{i}(\delta \phi )$ and ${\overset{ˆ}{a}}_{j}^{†}$ from left to right, ${\overset{ˆ}{a}}_{j}^{†}$ is transferred to the superposition state described by the sum of ${\overset{ˆ}{a}}_{k}^{†}{\left(D_{i}(\delta \phi )\right)}_{kj}$. Note that ${\overset{ˆ}{a}}_{k}^{†}$ is multiplied from the right-hand side [115] by the unitary transformation of ${\left(D_{i}(\delta \phi )\right)}_{kj}$. If we repeat to use Equation (15), we obtain

(17) $${\overset{ˆ}{D}}_{i}(\delta \phi ){\left({\overset{ˆ}{a}}_{j}^{†}\right)}^{n}={\left(\sum_{k}{\overset{ˆ}{a}}_{k}^{†}{\left(D_{i}(\delta \phi )\right)}_{kj}\right)}^{n}{\overset{ˆ}{D}}_{i}(\delta \phi ).$$

If we apply both sides of Equation (17) to a vacuum state, only the identity operation of **1** within ${\overset{ˆ}{D}}_{i}(\delta \phi )$ survives upon the application of ${\overset{ˆ}{D}}_{i}(\delta \phi )$ to |0〉, since ${\overset{ˆ}{a}}_{l}|0\rangle =0$. Then, we obtain

(18) $${\overset{ˆ}{D}}_{i}(\delta \phi ){\left({\overset{ˆ}{a}}_{j}^{†}\right)}^{n}|0\rangle ={\left(\sum_{k}{\overset{ˆ}{a}}_{k}^{†}{\left(D_{i}(\delta \phi )\right)}_{kj}\right)}^{n}|0\rangle .$$

This means that the SU(*N*) transformation of ${\overset{ˆ}{D}}_{i}(\delta \phi )$ corresponds to change ${\overset{ˆ}{a}}_{j}^{†}\to \sum_{k}{\overset{ˆ}{a}}_{k}^{†}{\left(D_{i}(\delta \phi )\right)}_{kj}$. By expanding the coherent state in Taylor series, we can apply Equation (18) to obtain

(19) $$\prod_{j}{\overset{ˆ}{D}}_{i}(\delta \phi )e^{{\overset{ˆ}{a}}_{j}^{†}\alpha_{j}}|0\rangle =\prod_{j}{\overset{ˆ}{D}}_{i}(\delta \phi )\sum_{n=0}^{\infty }\frac{1}{n!}{\left({\overset{ˆ}{a}}_{j}^{†}\alpha_{j}\right)}^{n}|0\rangle$$

(20) $$=\prod_{j}\sum_{n=0}^{\infty }\frac{1}{n!}{\left(\sum_{k}{\overset{ˆ}{a}}_{k}^{†}{\left(D_{i}(\delta \phi )\right)}_{kj}\alpha_{j}\right)}^{n}|0\rangle$$

(21) $$=e^{\sum_{kj}{\overset{ˆ}{a}}_{k}^{†}{\left(D_{i}(\delta \phi )\right)}_{kj}\alpha_{j}}|0\rangle$$

(22) $$=e^{\sum_{k}{\overset{ˆ}{a}}_{k}^{†}\alpha_{k}^{\prime }}|0\rangle ,$$

where we confirmed $\alpha_{k}^{\prime }=\sum_{j}{\left(D_{i}(\delta \phi )\right)}_{kj}\alpha_{j}$, which is the matrix form of Equation (5). It is also straightforward to confirm the norm of $\alpha^{\prime }$ is conserved upon the unitary transformation by $D_{i}(\delta \phi )$, as $\sum_{k}|\alpha_{k}^{\prime }{|}^{2}=\sum_{j}|\alpha_{j}{|}^{2}$. This proves Equation (4), such that the coherent state has SU(*N*) symmetry if the corresponding single-particle state has SU(*N*) degrees of freedom.

Historically, Schwinger employed the spin angular momentum for SU(2), known as Schwinger bosons [131], and applied to a Fock state with fixed number of bosons [131], [92], [132], [120]. If we apply ${\overset{ˆ}{A}}_{1}$ and ${\overset{ˆ}{A}}_{2}$ for a state to diagonalise ${\overset{ˆ}{A}}_{3}$, the numbers of bosons with up and down spin are changed. Consequently, the overlap with the original state vanishes after the applications of ${\overset{ˆ}{A}}_{1}$ and ${\overset{ˆ}{A}}_{2}$, such that expectation values for these operators vanish. The analysis is useful to understand the role of angular momentum for the transition of the bosonic state with fixed number of bosons [131], [92], [132], [120]. However, we cannot correlate the vanishing spin angular momentum with Stokes parameters to understand polarisation [87], [88], [89], [90], [91], [92], [133], [93], [94], [60], [61], [62], [104], [105], [106], [107], [108], [63], [64], [78], [81], which is the manifestation of spin of photons at a macroscopic scale. On the other hand, the coherent state in the present work is a superposition state among states with different number of photons, and we could confirm that the form of the coherent state is preserved as Equation (4) upon the SU(*N*) transformation of Equation (2).

### 2.3 Expectation values transferred in SO($N^{2}-1$) space

The generators ${\overset{ˆ}{A}}_{j}$ of the SU(*N*) unitary transformation [130][114][115][113][118][119][120], [121], [122], [123][78][48] is an Hermite matrix, whose expectation value,

(23) $$A_{j}\equiv \langle \alpha_{1},\cdots ,\alpha_{N}|{\overset{ˆ}{A}}_{j}|\alpha_{1},\cdots ,\alpha_{N}\rangle$$

(24) $$=\hbar \left(\alpha_{1}^{⁎},\cdots ,\alpha_{N}^{⁎}\right)A_{j}\left(\begin{array}{c} \alpha_{1}\\ \vdots \\ \alpha_{N} \end{array}\right)$$

(25) $$=\hbar \langle I|A_{j}|I\rangle ,$$

is real (R) and observable. It is important to recognise that the expectation value is calculated just like an expectation value for a single photon with the SU(*N*) symmetry, although many photons are involved in the coherent state of Equation (1). This means indistinguishable photons are coherently occupying the same SU(*N*) state. The calculated values in average angular momentum, $A={\left(A_{1},\cdots ,A_{N}\right)}^{t}$, are proportional to *ħ* and the number of photons (N) per second passing through a cross-sectional area perpendicular to a direction of propagation. Note the SU(*N*) wavefunction is normalised as $\langle I|I\rangle =\alpha^{†}\alpha =N$, where ^†^ stands for a complex conjugate operation.

We consider how $A_{i}$ is changed upon the SU(*N*) unitary transformation of ${\overset{ˆ}{D}}_{i}(\delta \phi )$, shown in Equation (2). We calculate

(26) $$A_{j}^{\prime }\equiv \langle \alpha_{1}^{\prime },\cdots ,\alpha_{N}^{\prime }|{\overset{ˆ}{A}}_{j}|\alpha_{1}^{\prime },\cdots ,\alpha_{N}^{\prime }\rangle$$

(27) $$=\hbar \alpha^{\prime †}A_{j}\alpha^{\prime }$$

(28) $$=\hbar \alpha^{†}D_{i}{\left(\delta \phi \right)}^{†}A_{j}D_{i}(\delta \phi )\alpha$$

(29) $$=\hbar \alpha^{†}e^{iA_{i}\frac{\delta \phi }{2}}A_{j}e^{-iA_{i}\frac{\delta \phi }{2}}\alpha$$

(30) $$=\hbar \langle I|e^{iA_{i}\frac{\delta \phi }{2}}A_{j}e^{-iA_{i}\frac{\delta \phi }{2}}|I\rangle .$$

Again, we use the Campbell-Baker-Hausdorff formula [91][92] of Equation (7) by putting $\overset{ˆ}{X}=iA_{i}(\delta \phi /2)$ and $\overset{ˆ}{Y}=A_{j}$. We successively obtain

(31) $$[\overset{ˆ}{X},\overset{ˆ}{Y}]=\sum_{k}f_{ijk}A_{k}\left(-\frac{\delta \phi }{2}\right)$$

(32) $$[\overset{ˆ}{X},[\overset{ˆ}{X},\overset{ˆ}{Y}]]=\sum_{k,k^{\prime }}f_{ijk}f_{ikk^{\prime }}A_{k}{\left(-\frac{\delta \phi }{2}\right)}^{2}$$

(33) $$[\overset{ˆ}{X},[\overset{ˆ}{X},[\overset{ˆ}{X},\overset{ˆ}{Y}]]]=\sum_{k,k^{\prime }k^{\prime \prime }}f_{ijk}f_{ikk^{\prime }}f_{ik^{\prime }k^{\prime \prime }}A_{k}{\left(-\frac{\delta \phi }{2}\right)}^{3},$$

and so on. Therefore, we obtain

(34) $$A_{j}^{\prime }=\sum_{k}[\delta_{jk}+{\left(F_{i}\right)}_{jk}\left(-\frac{\delta \phi }{2}\right)+\frac{1}{2}{\left(F_{i}^{2}\right)}_{jk}{\left(-\frac{\delta \phi }{2}\right)}^{2}+\frac{1}{3!}{\left(F_{i}^{3}\right)}_{jk}{\left(-\frac{\delta \phi }{2}\right)}^{3}+\cdots ]=\sum_{k}{\left(e^{-F_{i}\frac{\delta \phi }{2}}\right)}_{jk}A_{k},$$

where we have defined the adjoint operator $F_{i}$ from its matrix element of ${\left(F_{i}\right)}_{jk}=f_{ijk}$. Note that $e^{-F_{i}\frac{\delta \phi }{2}}$ is an $\left(N^{2}-1)\times (N^{2}-1\right)$ matrix and works as a rotational operator in SO($N^{2}-1$) space for expectation values.

As an application of this formalism, we consider spin angular momentum of coherent photons [104], [105], [106], [107], [108], [78], we should use the Pauli matrices of $\sigma_{i}=A_{i}$ (i=1,2,3) for SU(2), which satisfy the commutation relationship of $[\sigma_{i},\sigma_{j}]=2i\epsilon_{ijk}\sigma_{k}$ using the complete anti-symmetric Levi-Civita symbol $\epsilon_{ijk}$. In this case, we assume N=2 and $f_{ijk}=2\epsilon_{ijk}$ and the SU(2) wavefunction is ${\left(\alpha_{1},\alpha_{2}\right)}^{t}=\sqrt{N}{\left(e^{-i\theta /2}\cos(\phi /2),e^{i\theta /2}\sin(\phi /2)\right)}^{t}$ and we obtain the spin expectation values of ${\left(S_{1},S_{2},S_{3}\right)}^{t}\equiv {\left(A_{1},A_{2},A_{3}\right)}^{t}=\hbar N{\left(\sin\theta \cos\phi ,\sin\theta \sin\phi ,\cos\theta \right)}^{t}$ in agreement with Stokes parameters [104], [105], [106], [107], [108], [78]. This corresponds to the coherent state, whose spin is pointing to a direction, defined by the polar angle of *θ* and the azimuthal angle of *ϕ*. Then, we consider a rotation along the third axis to obtain the rotated spin expectation values

(35) $$S^{\prime }=\hbar N\left(\begin{array}{ccc} \cos(\delta \phi )\; & -\sin(\delta \phi )\; & 0\\ \sin(\delta \phi )\; & \cos(\delta \phi )\; & 0\\ 0\; & 0\; & 1 \end{array}\right)\left(\begin{array}{c} \sin\theta \cos\phi \\ \sin\theta \sin\phi \\ \cos\theta \end{array}\right)=\hbar N\left(\begin{array}{c} \sin\theta \cos(\phi +\delta \phi )\\ \sin\theta \sin(\phi +\delta \phi )\\ \cos\theta \end{array}\right),$$

which is expected rotation in SO(3). We can also confirm that it is required to rotate twice in SO(3) for preserving the geometric phase of the wavefunction after the SU(2) transformation by changing *δϕ* from 0 to 4*π*, since SU(2) is a two-fold coverage of SO(3), which is described by SU(2)/Z_2_ ≃ SO(3) using a cyclic group of order two, Z=2{0,1}. Consequently, the polarisation state is described on the Poincaré sphere, a sphere of two dimensional surface ($S^{2}$), whose radius of $S_{0}=\sqrt{S_{1}^{2}+S_{2}^{2}+S_{3}^{2}}=\hbar N$ is conserved upon the SU(2) transformation.

In general, the radius ($A_{0}$) of angular momentum (***A***) is conserved upon the SU(*N*) transformation. This can be confirmed from Equation (33) by calculating

(36) $$\sum_{j}{\left(A_{j}^{\prime }\right)}^{2}=\sum_{k,k^{\prime }}{\left(e^{-F_{i}\frac{\delta \phi }{2}}\right)}_{jk}A_{k}{\left(e^{-F_{i}\frac{\delta \phi }{2}}\right)}_{jk^{\prime }}A_{k^{\prime }}$$

(37) $$=\sum_{k,k^{\prime }}A_{k}{\left(e^{+F_{i}\frac{\delta \phi }{2}}\right)}_{kj}{\left(e^{-F_{i}\frac{\delta \phi }{2}}\right)}_{jk^{\prime }}A_{k^{\prime }}$$

(38) $$=\sum_{j}{\left(A_{j}\right)}^{2},$$

where we have used the anti-symmetric property of $f_{ijk}=-f_{ikj}$. This means that $A_{0}$ is independent on the SU(*N*) state and we just need to calculate

(39) $${\left(A_{0}\right)}^{2}=\hbar^{2}N^{2}\sum_{j}{\langle A_{j}\rangle }^{2}$$

for one of the SU(*N*) wavefunction, where we defined the quantum-mechanical average of 〈⋅〉=〈I|⋅|I〉/〈I|I〉 as usual [91][92]. According to the Cartan-Dykin formalism for the SU(*N*) transformation, we can classify whether $A_{j}$ (j=1,⋯,N) belongs to a diagonalised Cartan sub-algebra or not [115][113][118][119]. From the definition, Cartan operators are Hermite and observables. Thus, we can choose the basis states, which are eigenstates of Cartan operators. The corresponding eigenvalues are known as weights in mathematics [115][113][118][119]. The rest of the operators are ladder operators to increase or decrease the eigenvalues of the Cartan operators. Specifically, Cartan operators correspond to $A_{j}$ for $j=3,8,15,\cdots ,N^{2}-1$ (N≥2). We consider a state of $\alpha ={\left(1,0,\cdots ,0\right)}^{t}$, which gives vanishing expectation values for ladder operators, since ladder operators change the state to its orthogonal state. For spin this corresponds to $\langle \sigma_{1}\rangle =\langle \sigma_{2}\rangle =0$ for ladder operators of $\sigma_{\pm }=\sigma_{1}\pm i\sigma_{2}$, and for the Cartan operator we obtain $\langle \sigma_{3}\rangle =1$. Therefore, we just need to calculate expectation values for Cartan operators, which become $\langle A_{3}\rangle =1$, $\langle A_{8}\rangle =1/\sqrt{3}$, $\langle A_{15}\rangle =1/\sqrt{6}$, $\langle A_{24}\rangle =1/\sqrt{10}$, $\langle A_{35}\rangle =1/\sqrt{15}$, ⋯, and so on. In general, the Cartan operator of $A_{n^{2}-1}$ is defined to have (n−1) successive diagonal elements of one, followed by −(n−1) in the last diagonal element to be traceless, whose normalisation constant becomes $\sqrt{2/n(n-1)}$ to guarantee the trace of $\mathrm{tr}(A_{n}A_{n})=2$ in agreement with $\mathrm{tr}(\sigma_{i}^{2})=2$. As a result, we obtain $\langle A_{n^{2}-1}\rangle =\sqrt{2/n(n-1)}$ for $\alpha ={\left(1,0,\cdots ,0\right)}^{t}$. Therefore, the radius is obtained as $A_{0}=\hbar NA_{0}$, where $A_{0}$ is calculated as

(40) $${\left(A_{0}\right)}^{2}=\sum_{n=2}^{N}{\left(\langle A_{n^{2}-1}\rangle \right)}^{2}=\sum_{n=2}^{N}\frac{2}{n(n-1)}=2\sum_{n=2}^{N}\left(\frac{1}{n-1}-\frac{1}{n}\right)=2\frac{N-1}{N}.$$

For SU(2), SU(3), SU(4), SU(5), and SU(6), $A_{0}$ is 1, 4/3=1.3$\overset{˙}{3}$, 3/2=1.5, 8/5=1.6, and 5/3=1.6$\overset{˙}{6}$, respectively, and in the limit of N→∞ we obtain ${\left(A_{0}\right)}^{2}\to 2$. This means that $A_{0}$ increases from 1 to $\sqrt{2}$ as N→∞.

Note that SO($N^{2}-1$) space is much larger than SU(*N*) space for N≥3, such that the SU(*N*) state cannot cover the entire surface of the hypersphere of SO($N^{2}-1$) space for expectation values. This can be understood by recognising the topological space of the SU(*N*) wavefunction requires *N* complex numbers, as $\alpha \in C^{N}$, which reduces to $\alpha \in S_{C}^{N-1}=S^{2N-1}$ after the normalisation. Therefore, we need a maximum of 2N−1 real parameters. On the other hand, SO($N^{2}-1$) space corresponds to $S^{N^{2}-2}$, which corresponds to $N^{2}-2$ real parameters. In fact, we have previously shown that expectation values of generators of transformation in a SU(3) state are naturally embedded in SO(6) ≃ $S^{5}$, and they can also be embedded SO(5) ≃ $S^{4}$ if the axis is properly chosen, such that SO(8) space cannot be covered entirely. This is in contrast to the two-fold coverage of SU(2) for SO(3) ≃ $S^{2}$. Consequently, some areas of the hypersphere for N≥3 cannot be covered by SU(*N*) transformations, but conversely, SO($N^{2}-1$) space is more than enough to understand the nature of the SU(*N*) state, except for the U(1) factor for the geometrical phase [134], [135], [136], [137], [12].

### 2.4 SU(3) and SU(4) symmetry for coherent photons

As shown above, a hypersphere is required to describe all expectation values in a compact form. On the other hand, it is not possible to visualise the hypersphere in our three-dimensional space to get an intuitive picture. Instead, we can employ several Poincaré spheres [1], [2], [3], [4], [5], [6], [7], [8], [9], [10], [11], [12], [13], [14], [15], [16], [17], [18], [19], [20], [21], [22], [48] in SO(3) space, to show the expectation values among SU(2) states with spin and orbital angular momentum.

Specifically, we can assign $T_{1}=A_{1}=\sigma_{1}$, $T_{2}=A_{2}=\sigma_{2}$, and $T_{3}=A_{3}=\sigma_{3}$ for spin [87], [88], [89], [90], [91], [92], [133], [93], [94], [60], [61], [62], [63], [64], [78] or orbital angular momentum [3], [138][120], [121], [122], [123]. For higher-order SU(*N*) states (N≥3), we can always pick up two orthogonal states to form SU(2) sub-algebras [113], [115], [48]. For example, if we would like to discuss the superposition state between the first state of ${\left(1,0,0,\ldots \right)}^{t}$ and the third state of ${\left(0,0,1,\ldots \right)}^{t}$, we should employ $V_{1}=A_{4}$, $V_{2}=A_{5}$, and $V_{3}=A_{3}/2+\sqrt{3}A_{8}/2$ to describe the SU(2) state and their expectation values are shown on the Poincaré sphere with the SO(3) symmetry. Similarly, we can also consider the superposition state between the second state of ${\left(0,1,0,\ldots \right)}^{t}$ and the third state by $U_{1}=A_{6}$, $U_{2}=A_{7}$, and $U_{3}=-A_{3}/2+\sqrt{3}A_{8}/2$ to describe the SU(2) state and their expectation values are shown on the Poincaré sphere with the SO(3) symmetry. In this way, we can consider three SU(2) sub-algebras for SU(3) symmetry, using nine matrices of $T=(T_{1},T_{2},T_{3})$, $U=(U_{1},U_{2},U_{3})$, and $V=(V_{1},V_{2},V_{3})$. Note that we should have eight generators for SU(3) symmetry due to the traceless condition, such that the identity condition of $V_{3}=U_{3}+T_{3}$ must be satisfied. Consequently, we consider three Poincaré spheres for SU(3) states [48].

Similarly, for the SU(4) states, we should require six SU(2) sub-algebras to pick up two states among four orthogonal states [113], [115]. Three SU(2) algebras are exactly the same as those for SU(3) symmetry. In addition, we should define $W=(W_{1},W_{2},W_{3})=(A_{9},A_{10},A_{3}/2+A_{8}/(2\sqrt{3})+\sqrt{2}A_{15}/\sqrt{3})$, $X=(X_{1},X_{2},X_{3})=(A_{11},A_{12},-A_{3}/2+A_{8}/(2\sqrt{3})+\sqrt{2}A_{15}/\sqrt{3})$, and $Z=(Z_{1},Z_{2},Z_{3})=(A_{13},A_{14},-A_{8}/\sqrt{3}+\sqrt{2}A_{15}/\sqrt{3})$ [113], [115]. Here, we have defined eighteen generators, among which fifteen generators are independent. Therefore, we have two more identities to satisfy $W_{3}=T_{3}+X_{3}$ and $U_{3}=X_{3}-Z_{3}$ [113], [115]. We think these assignments of generators of transformations in SO(15) are useful to clarify the parameters of the higher order Poincaré spheres [1], [2], [3], [4], [5], [6], [7], [8], [9], [10], [11], [12], [13], [14], [15], [16], [17], [18], [19], [20], [21], [22], [48].

More generally, for the SU(*N*) states, we should consider N(N−1)/2 sub-algebras of SU(2), such that there exists N(N−1)/2 Poincaré spheres. In this case, we consider 3N(N−1)/2 generators, among which $N^{2}-1$ generators are independent. As a result, we should identify $3N(N-1)/2-N^{2}+1$ identities for eigenstates [113], [115].

**Figure 1:**
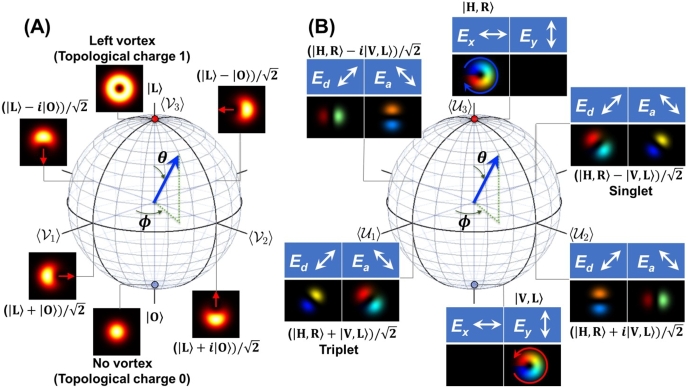
Higher-order Poincaré spheres for spin and orbital angular momentum. (A) SU(3) states to describe the coupling between left vortex (|L〉) and no-vortex (|O〉) states. The topological charge escapes from the core of the mode upon increasing the amplitude of |O〉, whose direction is controlled by the phase, shown by red arrows. (B) SU(4) states to describe the coupling between horizontally-polarised right vortex (|H,R〉) and vertically-polarised left vortex (|V, L〉) states. The superposition states along the equator of the Poincaré sphere are characterised by the optical dipoles, whose directions depend on the diagonal (*E*_d_) and anti-diagonal (*E*_a_) polarisation components.

The higher-order Poincaré spheres [1], [2], [3], [4], [5], [6], [7], [8], [9], [10], [11], [12], [13], [14], [15], [16], [17], [18], [19], [20], [21], [22] considered in this work for experiments are shown in Fig. 1. For SU(3) states [48], we discuss the superposition states between a twisted state and a no-twisted state for vertical polarisation (Fig. 1(A)). We show that the state is characterised by the dynamics of topological charge upon changing the phase and the amplitude of the SU(3) state. For SU(4) states, we consider the coupling among various spin and orbital angular momentum states and show that the classically entangled SU(4) states along the equator of the higher order Poincaré sphere is projected to be SU(2)×SU(2) states upon passing through the diagonal and anti-diagonal polarisers (Fig. 1(B)).

## 3 Experimental set-up

As discussed above, we need to prepare an SU(2) operator to realise an SU(*N*) state, since an SU(*N*) transformation can be constructed by combining several SU(2) operators [117], [118], [119], [113], [96], [110], [111], [112], [114], [115], [116], [48]. For example, arbitrary SU(3) operations can be achieved by two sets of SU(2) operators, since the Lie algebra of su(3) is rank two [113], [115], [48]. We have previously shown that an arbitrary SU(2) operation can be realised by a proposed Poincaré rotator using fibre optic experiments [83], [84], [139], [85]. Here, we have prepared a similar set-up for passive free space experiments. This allows us to check the proof-of-concept easily, and it is also useful to extend the Poincaré rotator to allow coupling among orthogonal twisted modes with spin-to-orbital conversion using vortex lenses [2], [3], [4], [5], [6], [7], [8], [9], [10], [11], [12], [13].

### 3.1 Poincaré rotator

**Figure 2:**
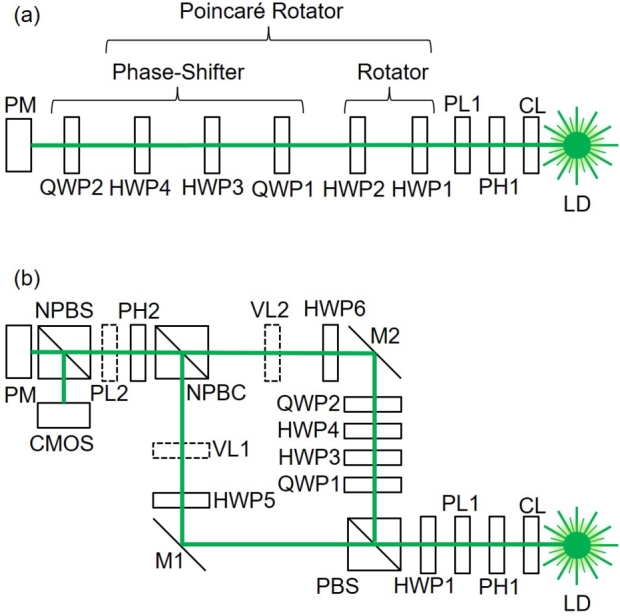
Experimental set up for passive Poincaré rotators. Abbreviations are as follows: LD: Laser Diode, CL: Collimator Lens, PH: Pin Hole, PL: Polariser, HWP: Half-Wave Plate, QWP: Quarter-Wave Plate, PBS: Polarisation Beam Splitter, NPBC: Non-Polarisation Beam Combiner, NPBS: Non-Polarisation Beam Splitter, M: Mirror, PM: Polarimeter, CMOS: Camera. (a) Poincaré rotator for polarisation. (b) Poincaré rotator for arbitrary orthogonal modes with and without a vortex. VL1, VL2, and PL2 are shown by dotted lines, indicating that we have conducted experiments both with and without these optical components.

The proposed Poincaré rotator in free space [83], [84], [139], [85] is shown in Fig. 2 (a). We have used a Diode-Pumped Solid State (DPSS) Laser-Diode (LD) with a wavelength of 532 nm, which was operated at the temperature of 20 ^∘^C under the constant current of 120 mA with the output power of ∼1 mW. The beam was collimated using a convex lens (a Collimator Lens, CL) with the focal length, *f*, of 100 mm. The beam shape was refined by using a Pin Hole (PH) with a diameter of 200 μm to make a Gaussian mode profile. The ray was passing through the polariser (PL) to make the polarisation state horizontal to an optical table with a vibration isolation.

Then, we have inserted into a Poincaré rotator [83], [84], [139], [85], which comprises from a rotator and a phase-shifter. The rotator is simply made of two successive operations of Half-Wave Plates (HWPs) [84]. The HWP2 was used upon rotating physically, such that its Fast Axis (FA) is aligned to a certain angle, measured from the horizontal direction. It is well known that the rotated HWP works as a pseudo-rotator [61], [62], which is actually a mirror reflection of the input polarisation state [78], [84]. The pseudo-rotator, associated with the corresponding mirror reflection, does not form a group [117], [118], [119], [113], [115], since two successive operations of mirror simply correspond to an identity operation, while it is required to rotate twice the angle of the pseudo-rotation for establishing a group. This issue was overcome by introducing additional HWP1 before operating by rotated HPW2 [78], [84]. The HWP1 was set its FA to align along the horizontal direction, which converts the pseudo-rotator to a pristine rotator to form a group for rotations [78], [84]. Therefore, we can construct an rotator of the polarisation state at an arbitrary rotation angle determined by a physical rotation angle of HWP2 [78], [84]. The rotator controls the relative amplitudes between horizontally-polarised and vertically-polarised states, such that it allows us to rotate the polarisation state on the Poincaré sphere along the azimuth direction in the $S_{1}$-$S_{2}$ plane. The proper rotator corresponds to the U(1) ≅ SO(2) operation, which becomes a crucial component to realise transformations in higher order SU(*N*).

It is important to be aware that the physical rotation angle of ΔΨ for HPW2 corresponds to the four times of the rotation of the polarisation state on the Poincaré sphere, 4ΔΨ, along the azimuthal direction [61], [62]. The factor of two is coming from the mirror reflection of the complex electric fields, and another factor of two is coming from the difference of angles of SU(2) wavefunctions and its expectation value [78], [84]. This factor of four is practically critical, since the uncertainty of physical rotation angle expands the deviation of the azimuthal angle on the Poincaré sphere from the expected value [61], [62], [78], [84].

Then, the ray, rotated by a rotator, is inserted in a phase-shifter (Fig. 2 (a)), which rotates the polarisation state in the $S_{2}$-$S_{3}$ plane on the Poincaré sphere. In order to realise a phase-shifter, we need to insert 2 Quarter-Wave Plates (QWPs) before and after the rotator. The first QWP1 is aligned along the anti-diagonal direction, which is rotated $-45^{\circ }$ from the horizontal axis, seen from the detector side [78]. This makes the $S_{2}$-$S_{3}$ plane to the $S_{1}$-$S_{2}$ plane, such that we can rotate the polarisation state using a rotator, made of HWP3 and HWP4. Finally, we can bring the rotated plane back to the original plane, by aligning the QWP2 to the diagonal direction, which is rotated $45^{\circ }$ from the horizontal axis [78], [84], and the polarisation states were measured by a polarimeter (PM).

### 3.2 Ladder operation to increment orbital angular momentum

We have realised an arbitrary rotation of spin for photons as polarisation states by the proposed Poincaré rotator [83], [84], [139], [85]. We will use the Poincaré rotator as for a device to allow other SU(2) operations for photons with and without orbital angular momentum. In order to realise a superposition state among orthogonal states with orbital angular momentum, we needed to extend the free space set-up to increment orbital angular momentum using vortex lenses [2], [3], [4], [5], [6], [7], [8], [9], [10], [11], [12], [13].

Fig. 2 (b) shows the experimental set up, used in this work. We used the same DPSS LD at 532 nm as the one used in Fig. 2 (a), and the ray is collimated by the same CL and a PH1. We used the PL1 to make the ray to the horizontally polarised state, and we used HWP1 for a rotator. Here, we removed HWP2, since there is no difference between a pseudo-rotator and a pristine rotator for a horizontally polarised state. The advantage to make a pristine rotator configuration of Fig. 2 (b) is that the rotator works for any input, regardless of the polarisation states, including unknown polarisation states [78].

Then, the ray was separated between horizontally polarised state and the vertically polarised state by a Polarisation Beam Splitter (PBS). The PBS works as a converter from spin states to orbital paths, such that we can separate orthogonal polarisation states. The ray with the horizontally polarised state is propagating to the left and reflected by the mirror (M1) to change the direction vertically. We inserted another HWP5, which can be rotated to switch from the horizontally polarised state to the vertically polarised state by setting its FA to diagonally or anti-diagonally. Alternatively, we can also keep the horizontal polarisation state simply by setting its FA horizontally or vertically. This allows us to choose one of the polarisation state before combining again by a Non-Polarisation Beam Combiner (NPBC). It was our option to include a vortex lens (VL1) or not, depending on whether we would like to increment the no-twisted state to the twisted state by a ladder operation of the orbital angular momentum [79], [138]. In our experiments, we tried both cases, such that VL1 in Fig. 2 (b) is shown by a dotted line. When we included VL1, we intended to generate a left vortex, with orbital angular momentum of *ħ* per photon, whose chirality is defined by seeing from the detector side [78], [79], [80], [83], [84], [139], [85], [48].

For the path reflected vertically at the PBS, we have inserted a phase-shifter, using QWP1, HWP3, HWP4, and QWP2, exactly the same as the set-up, shown in Fig. 2 (a). Here, only HWP4 was physically rotated to control the phase-shift, while QWP1, HWP3, and QWP2 were aligned to $-45^{\circ }$, 0, and $45^{\circ }$ directions, respectively, measured from the horizontal axis. Note that the vertical polarisation state is not changed during the propagation through the phase-shifter operation by QWP1, HWP3, HWP4, and QWP2, since the phase-shifter rotates the polarisation state on the Poincaré sphere along the $S_{1}$ axis, and the vertically polarised state is located on the axis at $S_{1}=-1$. Nevertheless, the phase-shift is actually accompanied to the vertically polarised state, which is observable in comparison with the other ray, going to M1. Then, the ray after the phase-shifter is reflected at the M2, followed by the HWP6, which could be rotated to change the polarisation state. We have an option whether we include VL2 or not, as before, depending on generating a vortex or not. When we included VL2, we intended to generate a right vortex, with orbital angular momentum of −*ħ* per photon.

The rays coming from M1 and M2 were combined by a NPBC, which was made of a polarisation independent combiner with the ratio of 50:50. Therefore, the half of the intensity was lost, leaving to the vertical direction, which is not shown in Fig. 2 (b) for simplicity. The combined ray was passing through PH2 with the same diameter of 200 μm with PH1. We examined the ray with and without PL2, which selects a certain polarisation state, depending on the direction of the polariser. Finally, the output ray was separated by a Non-Polarisation Beam Splitter (NPBS) to analyse both polarisation and far-field images, using a PM and a Complementary-Metal-Oxide-Semiconductor (CMOS) camera, respectively.

Here, we must be careful for the change of spin and orbital angular momentum states, upon applications of mirrors [93], [94], [60], [61], [62], [63], [64], [133], [78]. The mirror operation effectively changes the signs of axes of *x* and *z*, while keeping the *y* axis the same. This could be easily understood, if we consider the total mirror reflection for the propagation against *z*, which will reflect the ray back to −*z*, such that the propagation direction is opposite and the right-handed *x*, *y*, and *z*, coordinate must be rotated $180^{\circ }$ along the *y* axis. In this coordinate, $S_{2}$ and $S_{3}$ should change sign upon the reflection, while $S_{1}$ should be the same [78], [82].

Another choice of the frame after the reflection is to swap *x* and *y*, while changing the sign of *z*, which is suitable to see the duality between complex conjugated states [82]. For the polarisation operation using su(2) operators of Pauli matrices $\overset{ˆ}{\sigma }=({\overset{ˆ}{\sigma }}_{1},{\overset{ˆ}{\sigma }}_{2},{\overset{ˆ}{\sigma }}_{3})$, this corresponds to use a complex conjugate representation by a mirror reflection of $\overset{ˆ}{\sigma }\to \overset{ˆ}{\overset{¯}{\sigma }}=-{\overset{ˆ}{\sigma }}^{⁎}=(-{\overset{ˆ}{\sigma }}_{1}^{⁎},-{\overset{ˆ}{\sigma }}_{2}^{⁎},-{\overset{ˆ}{\sigma }}_{3}^{⁎})=(-{\overset{ˆ}{\sigma }}_{1},{\overset{ˆ}{\sigma }}_{2},-{\overset{ˆ}{\sigma }}_{3})$ [115], [78], [82]. The same sign changes are also applicable to the twisted states, since the mirrors change the coordinate and the chirality is changed. Thus, for the SU(3) states, su(3) operators of ${\overset{ˆ}{\lambda }}_{i}$ (i=1, ⋯, 8) is reflected to be its complex conjugate ${\overset{ˆ}{\overset{¯}{\lambda }}}_{i}=-{\overset{ˆ}{\lambda }}_{i}^{⁎}$, and the wavefunction changes sign, accordingly [78], [48].

In our set-up, shown on Fig. 2 (b), the ray, going to the M1 with the horizontally polarised state, is reflected twice at M1 and NPBC to enter the PM, such that the twice of mirror operations will not change the polarisation state. Similarly, for the ray, going to M2 with the vertically polarised state, is reflected at the PBS and M2, such that no change of the polarisation state is expected for the ray entering into the PM. Here, it is very important to make sure that the number of mirror reflections should be the same for both paths. It is also allowed to have an even number of difference among different paths, but an odd number will mix rays, whose polarisation states are defined in a different coordinate and the analysis of polarisation states will be more difficult. For the ray going to a CMOS camera, we have an additional one more reflection at the NPBS, such that the polarisation state at the CMOS camera is the mirror reflected state of that at the PM. For the left vortex generated at VL1 will be kept left at the CMOS camera, since two mirror reflections are expected at the NPBC and the NPBS. On the other hand, only one reflection at NPBS is required for the twisted state generated at VL2 to entering into the CMOS camera. Therefore, we just need to generate the same left vortex at the VL2 for expecting the vortex to be the right vortex at the CMOS camera. This is absolutely fine, since the left vortex generated at VL1 will become the right vortex after the reflection at NPBC, such that after passing through NPBC, both left and right twisted states are combined to form one ray.

## 4 Experimental results and discussions

### 4.1 Polarisation controlled by Poincaré rotator

First, we used the experimental set-up of Fig. 2 (a), and confirmed the expected operations as a passive Poincaré rotator using a green LD. We show the rotator operation of the Poincaré operator along the equator of the Poincaré sphere. In this experiment, we have set the physical rotation angle (ΔΨ) of HWP2 at every 5^∘^, while other plates were kept fixed. We confirmed that the polarisation states were transferred upon rotating HWP2, as expected from the SU(2) theory for spin states of photons [78], [48].

**Figure 3:**
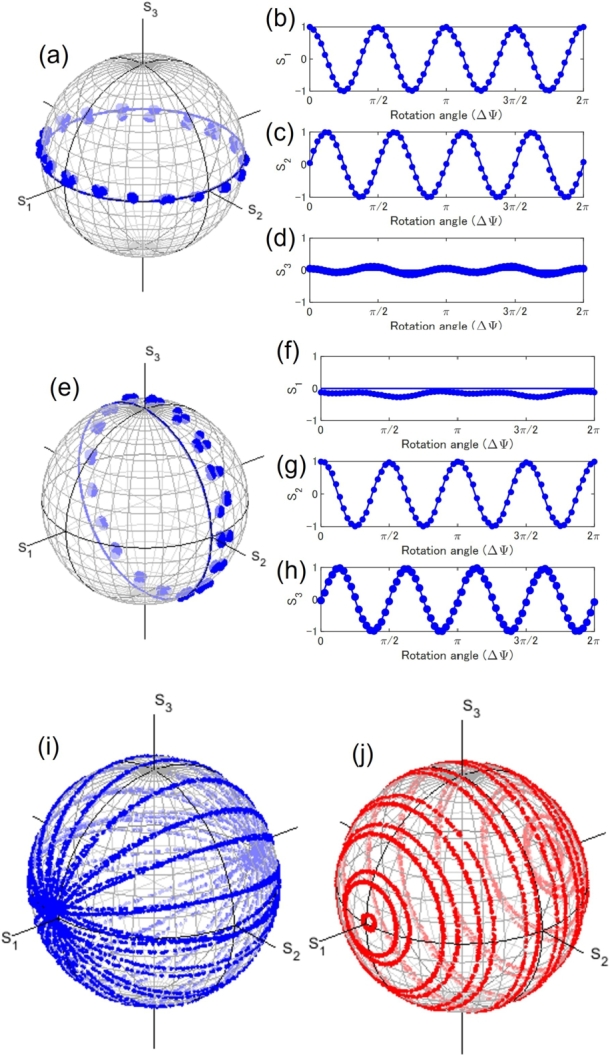
Poincaré rotator. Rotator operation of the Poincaré rotator, shown on (a) Poincaré sphere. Stokes parameters of (b) *S*_1_, (c) *S*_2_, and (d) *S*_3_ are also shown. We have inserted a horizontally polarised ray into the Poincaré rotator, and we have fixed the phase-shift to allow rotation of the polarisation states along the equator of the Poincaré sphere. At the zero physical rotation angle, ΔΨ, the polarisation state was not changed, while the polarisation state was rotated along the anti-clock-wise direction (left direction), seen from the top of the *S*_3_ axis. The polarisation states were rotated 4 times upon the physical rotation of the half-wave-plates, as expected from the SU(2) theory for spin states of coherent photons. Phase-Shifter operation of the Poincaré rotator, as shown on (e) Poincaré sphere and by Stokes parameters of (f) *S*_1_, (g) *S*_2_, and (h) *S*_3_. We found deviations from theoretical lines, which are considered to be within the expected errors of ∼10^∘^. Poincaré rotator operations as (i) a rotator and (j) a phase-shifter. The Stokes parameters were obtained by rotating corresponding wave-plates for 30 s, which were recorded by the polarimeter.

The rotator operation is shown on Figs. 3 (a)-(d). Similarly, we also confirmed the phase-shifter operation, as shown in Figs. 3 (e)-(h). Here, we rotated HWP2 to align its FA to $\pi /8=22.5^{\circ }$ form the horizontal direction to convert the horizontal input to the diagonally polarised state, located at $S_{2}=1$ before entering into the phase-shifter, Then, we have physically rotated HWP4, while other waveplates were kept fixed. In this case, the phase-shifter rotated the polarisation state on the Poincaré sphere in the $S_{2}$-$S_{3}$ plane.

The experimental data is consistent with this theoretical expectations shown by lines. However, we found remarkable deviations, corresponding to the angles of $∼10^{\circ }$, which are expected from the waveplates used in this experiment. Our waveplates have deviations of around a few degree at the wavelength of 532 nm, and the physical rotation of wave-plates would also induce uncertainty of $∼1^{\circ }$. Moreover, the rotation angle on the Poincaré sphere is four times larger than the physical rotation angle (δϕ=4ΔΨ), such that the errors of the angles also increase four times [61], [62], [78].

We also operated the Poincaré rotator by using both rotator and phase-shifter angles, as shown in Figs. 3 (i) and (j). For the rotator operation of Fig. 3 (i), we fixed the physical rotation angle for the phase-shifter at every $5^{\circ }$, while we have continuously rotated HWP2 for 30 s by hands and recorded Stokes parameters. We confirmed expected rotations for horizontal-vertical bases [78], [139], since the north and the south poles are located at $S_{1}=1$ and $S_{1}=-1$, respectively, for fixed phase-shifts, whose trajectories are similar to the longitude for the earth. On the other hand, we also confirmed the phase-shifter rotations upon the fixed rotator angles. We fixed the physical rotation angle of HWP2 at every $5^{\circ }$, while we have continuously rotated HWP4 by hands for 30 s and recorded by the PM. As shown in Fig. 3 (j), the rotation plane is always parallel to the $S_{2}$-$S_{3}$ plane, since the phases between horizontal and vertical states are defined without changing the amplitudes, which corresponds to keep the $S_{1}$ value, when we used the horizontal and vertical states as orthogonal bases [78], [139]. The trajectory in this case is similar to the latitude for the earth.

### 4.2 Poincaré rotator for orbital angular momentum

Next, we proceed to apply our simple experimental set-up for realising an SU(2) superposition state for orbital angular momentum [2], [3], [4], [5], [6], [7], [8], [9], [10], [11], [12], [48]. Here, we have introduced both VL1 and VL2 to induce both left- and right-twisted states to realise an arbitrary superposition state by our Poincaré rotator. We have not inserted PL2, for the moment, and HWP5 was set to align its FA to the $45^{\circ }$-rotated direction, measured from the horizontal axis. This corresponds to convert the horizontally polarised state for the ray, passing to M1, to the vertically polarised state. On the other hand, the FA of HWP6 was aligned to be horizontal, keeping the vertically polarised state for the ray, passing to M2. Therefore, both rays are in the vertically polarised state, such that we have effectively converted the spin degree of freedom to the orbital angular momentum for controlling amplitudes and the phase of left- and right-twisted states. We have generated the same magnitude of the topological charge of one for both left- and right-twisted states. The amplitudes between left- and right-twisted states were controlled by HWP1 to change the splitting ratio at PBS. On the other hand, the phase was controlled by the phase-shifter, made of QWP1, HWP3, HWP4, and QWP2. It must be mentioned that we have not controlled a global phase, solely by the difference of physical length upon propagation. It is not impossible to control the distance by using a stepping motor to control the distance with a resolution of the order of a few nm per step. In stead, we controlled the phase by the phase-shifter, such that the origin of the phase is not coincides with the physical rotation angle of HWP4, measured from the horizontal axis.

**Figure 4:**
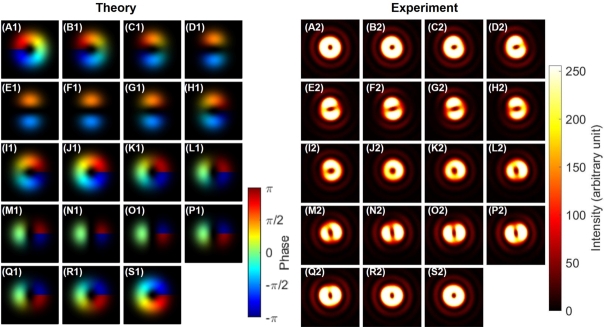
Rotator operation of Poincaré rotator for the orbital angular momentum. (A1)-(S1) theoretical calculations. (A2)-(S2) Experimental data. The half wave plate was physically rotated from (A) 0^∘^ to (S) 90^∘^ with a step of 5^∘^, which is enough to rotate from (A) the left vortex, via (J) the right vortex, back to (S) the left vortex, for circulating the Poincaré sphere. Dipole images were seen for (E)-(H) and for (M)-(P), which are orthogonal each other for the direction of the dipole.

As shown in Fig. 4, we observed expected SU(2) states with orbital angular momentum [2], [3], [4], [5], [6], [7], [8], [9], [10], [11], [12], [48] by rotating HWP1 as a rotator. We have shown both theoretical calculations (Figs. 4 A1-S1) and experimental results (Figs. 4 A2-S2). For the theoretical calculations, intensities are shown by the contrast, while the phase profiles are also shown by the colour profile. For experimental data, we could observe only intensity profiles. First, we confirmed a doughnut-like image of twisted states at Figs. 4 (A), (J), and (S), as expected for topological charge, stored at the centre of the mode [2], [3], [4], [5], [6], [7], [8], [9], [10], [11], [12], [79], [80]. We could also clearly confirm the dipole structures, realised by the superposition of left- and right-twisted states, as shown in Figs. 4 (E)-(H) and (M)-(P), which are orthogonal to each other. This means that superposition states of orthogonal orbital angular momentum states were successfully formed, and SU(2) states of the orbital angular momentum can be controlled along the equator of the sphere in the $\langle T_{1}\rangle$-$\langle T_{2}\rangle$ plane. We needed to rotate HWP1 for $90^{\circ }$ to realise one rotation in the SO(3) space, which was exactly the same as the rotation angle required for polarisation states.

**Figure 5:**
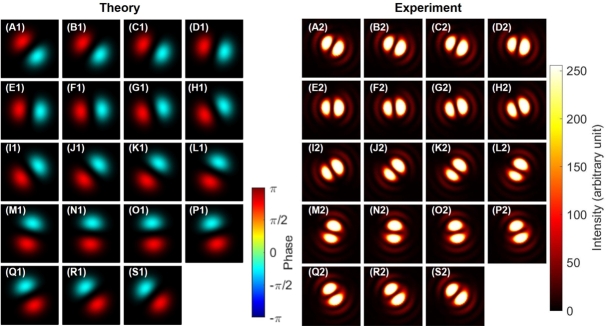
Phase-shifter operation of Poincaré rotator for orbital angular momentum. (A1)-(S1) theoretical calculations. (A2)-(S2) Experimental data. The half-wave plate was physically rotated from (A) 0^∘^ to (S) 90^∘^ with a step of 5^∘^, to realise the phase-shift up to *π*. Note that the dipoles were rotated upon the rotations the half-wave plate without changing the shape. The physical rotation of 45^∘^ is required to rotate the dipole for 90^∘^, which corresponds to change the state orthogonal to each other, corresponding to the 180^∘^-rotation on the Poincaré sphere.

Next, we confirmed the phase-shifter operation of the Poincaré rotator for orbital angular momentum states, which corresponds to the transformation in the $\langle T_{2}\rangle$-$\langle T_{3}\rangle$ plane. We set HWP1 to align its FA to $22.5^{\circ }$ from the horizontal direction, and we observed the dipole by the CMOS camera. This allows to split the ray into two rays with the 50:50 splitting ratio at the PBS. Then, we have rotated HWP4 to control the phase between left- and right-twisted states. As shown in Figs. 5 (A1)-(S1) and Figs. 5 (A2)-(S2), we observed the rotation of the dipole, upon rotating HWP4. In other words, we could identify the phase, from images of the dipole. Here, we must be careful about the definition of the phase. As explained above, we have not controlled the global phase, such that the orientation of the dipole at the zero-physical rotation of HWP4, as defined for the alignment of its FA to the horizontal direction, was not properly designed to align to a specific direction (Fig. 5 (A)). We can renormalise the phase, after observing the dipole image, to compensate the global phase, if we want.

The other interesting aspect on the phase is the relationship between the amount of rotation and the dipole image. If we carefully track the images from Figs. 5 (A1)-(S1) and Figs. 5 (A2)-(S2), one can identify the dipole is just rotating $180^{\circ }$ upon the physical rotation of $90^{\circ }$ for HWP4. This will bring one of two bright regions in the dipole image of Fig. 5 (A), located at the right-down region, to the other place of the left-up region in the dipole image of Fig. 5 (S). In the images, we could only recognise the intensities, but it is widely known that the phase of the dipole is opposite among two bright high-intensity regions. This means that the phase is opposite between the dipole of Fig. 5 (A) and the dipole of Fig. 5 (S), while average angular momentum defined on the SO(3) Poincaré sphere must be the same. This is indeed the manifestation of the two-fold coverage of SU(2) for SO(3), as mathematically known as $\mathrm{SU}(2)/S^{0}≅\mathrm{SU}(2)/Z_{2}≅\mathrm{SO}(3)$ [118], [119], [113], [115], [48]. Physically, expectation values often dismiss the geometrical phase information, such that we must be careful for their important roles [134], [135], [136], [137], [12].

### 4.3 Interference fringes

**Figure 6:**
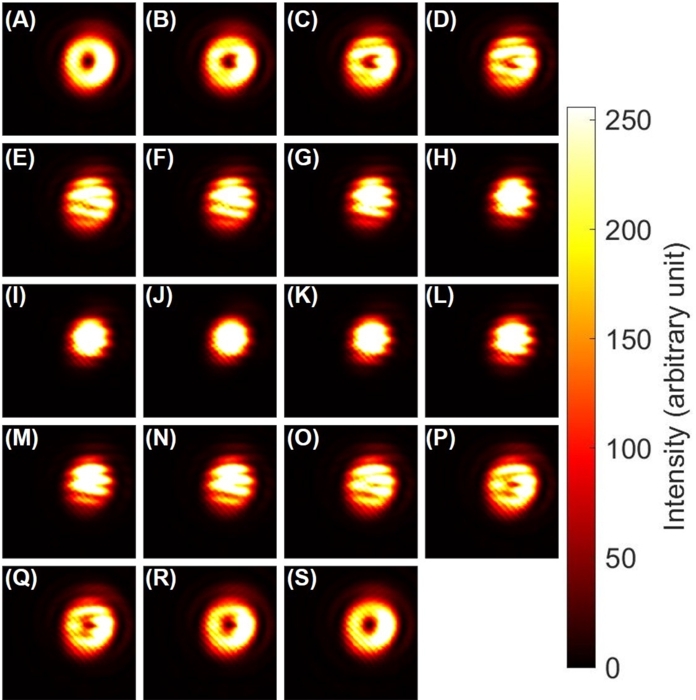
Interference fringes between the left twisted state and Gaussian state with the intensional miss-alignment between rays. The ratio of the intensities were controlled by rotator operations of Poincaré rotator upon rotating the half-wave plate from (A) 0^∘^ to (S) 90^∘^ with a step of 5^∘^. Far-field images of (A) and (S) a pure left-twisted beam, and (J) a Gaussian beam are shown, while we see continuous changes of interference fringes between these images.

After confirming the expected operation of Poincaré rotator for orbital angular momentum, we have checked interference fringes [13], [140], [141] between a twisted state and a non-vortex state. In order to observe the interference, we have removed VL2, while keeping the VL1, and we have intentionally miss-aligned for the ray with the left vortex and the ray without a vortex. After confirming the interference with equal intensities by the 50:50 splitting at the PBS, we have systematically changed the splitting by our rotator (Figs. 6 (A)-(S)).

Suppose if we have not used VL1 at all, we should just see continuous lines of interfere patterns for the superposition of two rays due to the miss-alignment which induces both constructive and destructive interference. The lines should be looked like a river to show the profile of the continuous phase as the intensity profile. On the other hand, if we have a topological charge, which induces 2*π* change of the phase around it, the number of interface fringes is different for the amount of the topological charge [13], [140], [141].

In fact, as confirmed in Figs. 6 (C), (D), (P) and (Q), we can recognise that one of the high intensity region is terminated at the centre of the topological charge, which looked like a fork. This confirms that we have generated a vortex of photons, whose topological charge, corresponding to the winding number of one [2], [13], [80].

**Figure 7:**
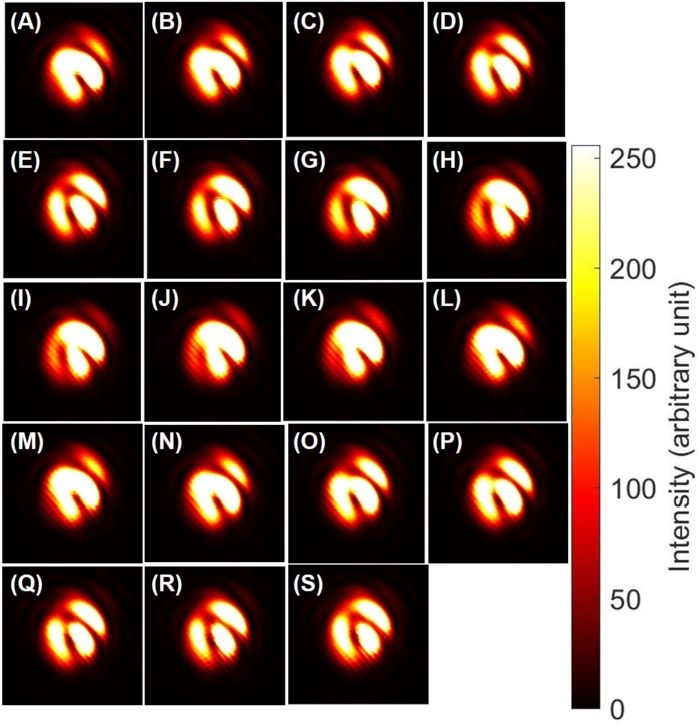
Interference fringes between the left twisted state and Gaussian state, upon changing the phase by the Poincaré rotator. The phases were controlled by phase-shifter operations of upon rotating the half-wave plate from (A) 0^∘^ to (S) 90^∘^ with a step of 5^∘^.

We have also observed the interference fringes, upon controlling the phase by the Poincaré rotator (Figs. 7 (A)-(S)). In this experiment, we have fixed the splitting ratio of 50:50 at the PBS, and we have changed the rotation angle of HWP4. We see that the interference fringes were continuously changed upon the phase-shifts (Figs. 7 (A)-(S)). It can also be recognised that the river-like fringe patterns were not affected significantly upon the phase-shifts, since the majority of the interference image is determined by the alignment between two rays. Consequently, the topological charge, which is trapped at the centre of the mode at Fig. 6 (A), could move through the river-like interference fringes to disappear to be a pure Gaussian mode at Fig. 6 (J). In other words, the river-like interference fringes were pined at the fixed position to determine the boundary conditions for the motion of the topological charge.

### 4.4 Topological colour-charged states

For SU(3) states, we consider the coupling between a twisted state and a no-twisted state under properly aligned conditions. In this case, we realised coupling among three orthogonal states, a left-twisted state (|L〉), a right-twisted state (|R〉), and a standard Gaussian beam with no vortex (|O〉), while the polarisation state was vertical. This corresponds to an SU(3) state, which is equivalent to assign topological colour charge to the orthogonal modes [48]. We have theoretically analysed the SU(3) states and found that two sets of SU(2) rotations can cover the whole Hilbert space, since the su(3) algebra is rank two [113], [115], [48]. We have already confirmed one set of such SU(2) transformations for coupling between |L〉 and |R〉, as shown in Figure 4, Figure 5. As a next step, we show SU(2) transformation for coupling between |L〉 and |O〉.

**Figure 8:**
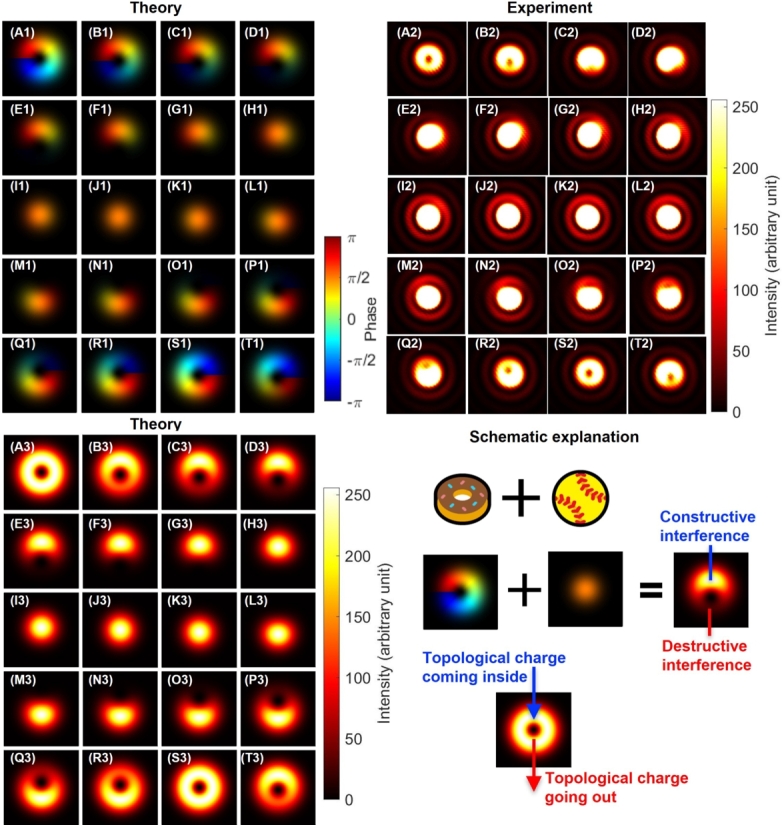
Superposition states between a left-twisted state and no twisted state. (A1)-(T1) theoretical calculations on phases. (A2)-(T2) Experimental data. (A3)-(T3) theoretical calculations on intensities. The amplitudes were controlled by rotating half-waveplates with the amount of (A) 0^∘^ to (T) 95^∘^ with a step of 5^∘^. The polarisation state was fixed to be vertical for all images. (A) and (S) a purely left-twisted state, and (j) a purely Gaussian state. Inset shows schematic explanations. The superposition state between a vortex and no twisted state produces both constructive and destructive regions in the ring, which breaks the ring of the vortex. Consequently, the topological charges escaped from being trapped inside the mode, as seen from (A)-(D) and (Q)-(T).

This corresponds to make a superposition state among states with different topological charge which must be orthogonal to each other. The topological charge must be robustly protected against the deformation of the mode, while a standard Gaussian mode does not contain the topological charge, such that the topological charge must be disappeared upon changing the relative amplitudes. The mode profile with a topological charge is similar to the shape of a doughnut, while the mode shape of the Gaussian mode is similar to a ball (Figs. 8 (A1)-(T1), Figs. 8 (A2)-(T2), and Figs. 8 (A3)-(T3)). Our question was how a doughnut could be changed to be a ball, or *vice versa*, which should not be achieved by topologically continuous deformation of a classical object. These two modes are orthogonal to each other such that we can distinguish them and realise the quantum superposition state among them to allow conversion from one to the other by changing the amplitudes.

The answer to this question is shown in Figs. 8 (A1)-(T1), Figs. 8 (A2)-(T2), and Figs. 8 (A3)-(T3). The topological charge moved to escape from being trapped inside the core of the mode (Visualization 1). In fact, the standard mathematical constraint on preventing to cut the doughnut is not applicable to our experiments, and the topological charge could cut the edge to leave from the core (Figs. 6 (C2) and (Q2)). In fact, upon changing the relative amplitudes between |L〉 and |O〉, some region of the mode will be enhanced due to the constructive interference, while the other region will be reduced by the destructive interference to cut the edge where the topological charge can escape (Figs. 8 (A1)-(T1)). This corresponds to the SU(3) transformation in the $\langle V_{2}\rangle$-$\langle V_{3}\rangle$ plane (Fig. 1 (A)).

The cut-line position is determined by the relative phase, which is not pinned by the interference fridges, shown in Figure 6, Figure 7. In the properly aligned set-up, there is no interference fringe any more, such that the position for the topological charge to escape must be controlled by the phase-shifter, due to the rotational symmetry of the mode (Figs. 8 (A1)-(T1), Figs. 8 (A2)-(T2), and Figs. 8 (A3)-(T3)).

### 4.5 Rotation of topological charge

**Figure 9:**
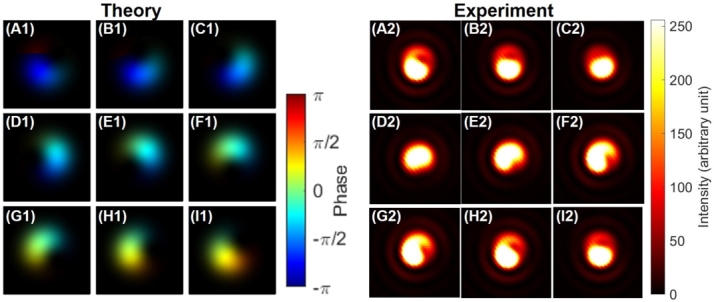
Superposition states between a left-twisted state and no twisted state, controlled by the phase-shifter. (A1)-(I1) theoretical calculations. (A2)-(I2) Experimental data. The phase was controlled by rotating half-wave plates with the amount of (A) 0^∘^ to (I) 180^∘^ with a step of 22.5^∘^. The amplitudes were fixed to be 50:50, separated at the polarisation beam splitter, while the polarisation was set to be vertical for both rays. The topological charge has just escaped at the edge, whose position was rotated upon the phase-shifts.

In order to see the change upon the phase-shifts, we have fixed the splitting at the PBS to be 50:50, and controlled the phase-shifts by rotating HWP4, as shown in Figs. 9 (A1)-(I1) and Figs. 9 (A2)-(I2).

This corresponds to the SU(3) transformation in the $\langle V_{1}\rangle$-$\langle V_{2}\rangle$ plane (Fig. 1 (A)). We see that the topological charge has just escaped from the edge, and the position seems to be rotated upon changing the phase. In particular, it should be recognised, by comparing Figs. 9 (A) and (I), that the $180^{\circ }$ rotation is required to rotate the bright region at the bottom of the superposition state. This is again coming from twofold coverage of SU(2) to SO(3), and it is required to rotate twice on the Poincaré sphere to bring the phase back to the original state. This is the same as the rotation of the polarisation state, which makes the horizontally polarised electric field $\left(E_{x},E_{y})=(E_{0},0\right)$ to $\left(-E_{0},0\right)$ upon 1 rotation on the Poincaré sphere, while rotations of twice is required to be $\left(E_{0},0\right)$. It is usually difficult to see the phase difference in the polarisation state, because the spin expectation values are the same for both states with the opposite phase. For the present case of the superposition state between |L〉 and |O〉, we can easily see the difference of the phase between Figs. 9 (A) and (E), since the interfere converts the phase difference to the intensity profile.

**Figure 10:**
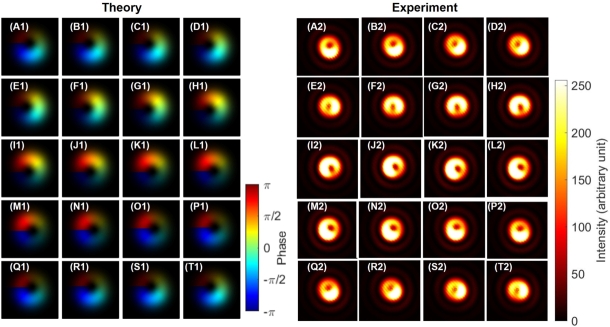
Superposition states between a left-twisted state and no twisted state, controlled by the phase-shifter. (A1)-(T1) theoretical calculations. (A2)-(T2) Experimental data. The phase was controlled by rotating half-wave plates with the amount of (A) 0^∘^ to (T) 190^∘^ with a step of 10^∘^. The topological charge is rotating along the anti-clock-wise direction upon increasing the phase-shift, which is expected for the operation of the phase-shifter using Poincaré rotator.

On the other hand, it was less clear to see how the topological charge was affected by the phase control from images shown in Figs. 10 (A1)-(T1) and Figs. 10 (A2)-(T2). Then, we have changed the amplitude by setting HWP1 to align its FA to $10^{\circ }$ from the horizontal direction, and controlled the phase upon rotating HWP4. As shown in Fig. 10, in this case, we could still see topological charge, located inside the mode, while the position of the topological charge is actually changed upon changing the phase-shift. The rotation is along the anti-clock-wise direction, which is the same as that for the dipole upon the phase-shift (Fig. 5).

### 4.6 Photonic singlet and triplet states

Finally, we examined SU(4) states and its projection to SU(2)×SU(2), without using the Gaussian mode without a vortex. Here, we consider two orthogonal polarisation states of horizontal and vertical states as bases for spin, and we also employ left and right twisted states as bases for orbital angular momentum. Therefore, we have four basis states, ${{|H\rangle }_{s}|L\rangle }_{o}$, ${{|H\rangle }_{s}|R\rangle }_{o}$, ${{|V\rangle }_{s}|L\rangle }_{o}$, ${{|V\rangle }_{s}|R\rangle }_{o}$, where s and o stand for spin and orbital angular momentum, respectively. In the SU(2) states of polarisation [87], [88], [89], [90], [91], [92], [133], [93], [94], [60], [61], [62], [63], [64], [78], we can choose the bases to horizontal and vertical states, rather than left and right circularly polarised states. In the horizontal and vertical bases, and the quantisation axis for the ${\overset{ˆ}{\sigma }}_{3}$ operator is attributed to the $S_{1}$ axis [78], such that we will assign spin up and down states as ${{|H\rangle }_{s}=|↑\;\rangle }_{s}$ and ${{|V\rangle }_{s}=|↓\;\rangle }_{s}$. Similarly, for orbital angular momentum, we assign ${{|L\rangle }_{o}=|↑\;\rangle }_{o}$ and ${{|R\rangle }_{o}=|↓\;\rangle }_{o}$. Consequently, we have defined four orthogonal states, as ${{{{|\;H\;\rangle }_{s}|\;L\;\rangle }_{o}=|↑\;\rangle }_{s}|↑\;\rangle }_{o}={\left(1,0,0,0\right)}^{t}$, ${{{{|\;H\;\rangle }_{s}|\;R\;\rangle }_{o}=|↑\;\rangle }_{s}|↓\;\rangle }_{o}={\left(0,1,0,0\right)}^{t}$, ${{{{|\;V\;\rangle }_{s}|\;L\;\rangle }_{o}=|↓\;\rangle }_{s}|↑\;\rangle }_{o}={\left(0,0,1,0\right)}^{t}$, and ${{{{|\;V\;\rangle }_{s}|\;R\;\rangle }_{o}=|↓\;\rangle }_{s}|↓\;\rangle }_{o}={\left(0,0,0,1\right)}^{t}$, which span the Hilbert space for SU(4) states since we can realise an arbitrary superposition state with variable phases and amplitudes among preferred combinations of these four states.

In fact, we have already confirmed that our Poincaré rotator creates an arbitrary SU(2) states for polarisation (Fig. 3), and we have also confirmed that the Poincaré rotator together with vortex lenses can generate an arbitrary SU(2) states for orbital angular momentum (Figure 4, Figure 5). However, as far as we are restricted to controlling polarisation states for fixed orbital angular momentum or to controlling orbital angular momentum for fixed polarisation states, the Hilbert space is limited in the direct product space of SU(2)×SU(2) states. In order to realise non-trivial SU(4) states, we need to couple ${{|↑\;\rangle }_{s}|↑\;\rangle }_{o}$ and ${{|↓\;\rangle }_{s}|↓\;\rangle }_{o}$, or to couple ${{|↑\;\rangle }_{s}|↓\;\rangle }_{o}$ and ${{|↓\;\rangle }_{s}|↑\;\rangle }_{o}$. The most famous states for such combinations would be the singlet state, $|\;\mathrm{Singlet}\;\rangle ={{{{(|↑\;\rangle }_{s}|↓\;\rangle }_{o}-|↓\;\rangle }_{s}|↑\;\rangle }_{o})/\sqrt{2}$, and the triplet state, $|\;\mathrm{Triplet}\;\rangle ={{{{(|↑\;\rangle }_{s}|↓\;\rangle }_{o}+|↓\;\rangle }_{s}|↑\;\rangle }_{o})/\sqrt{2}$, together with ${{|↑\;\rangle }_{s}|↑\;\rangle }_{o}$ and ${{|↓\;\rangle }_{s}|↓\;\rangle }_{o}$. In other words, we realise the coupling of spin and orbital angular momentum for classical entanglement [23], [26], [27], [28], [14], [15], [29], [30], and control the Clebsch-Gordan coefficients [91], [92]. The singlet and triplet states are also well-known as vector vortex beams [142], [15], [7], [47], and we provide a method to control the phase and amplitudes simply by rotating waveplates.

In our experiments, we used spiral vortex lenses (both VL1 and VL2 in Fig. 2) to generate left and right twisted states, respectively. We have aligned HWP5 to align its FA to the $45^{\circ }$ direction from the horizontal axis for rotating the polarisation from the horizontal state to the vertical state. Therefore, we have generated ${{|↓\;\rangle }_{s}|↑\;\rangle }_{o}$ for the ray going to M1. Similarly, we have aligned HWP6 to align its FA to the $45^{\circ }$ direction from the horizontal axis for rotating the polarisation from the vertical state to the horizontal state. This allowed us to generate ${{|↑\;\rangle }_{s}|↓\;\rangle }_{o}$ for the ray, going to the M2. We can control the phase between ${{|↑\;\rangle }_{s}|↓\;\rangle }_{o}$ and ${{|↓\;\rangle }_{s}|↑\;\rangle }_{o}$ by the phase-shifter, comprising of HWPs and Quarter-Wave-Plates (QWPs) of QWP1, HWP3, HWP4, and QWP2. For the phase-shift, we just need to rotate the HWP4, physically. The relative amplitudes were controlled by the rotator, such that we used HWP1 to set the 50:50 splitting at the PBS. Our experimental set-up allows us to realise both singlet and triplet states for coherent photons.

There was a practical challenge, however, to confirm the successful realisation of the singlet and triplet states since both left and right twisted states provide indistinguishable images at the CMOS camera. The singlet and triplet states mean that we can identify the difference in phase, once we identify the polarisation, but again the information on the phase could be disappeared in images.

These issues could be simply overcome by changing bases from horizontal and vertical bases to diagonal and anti-diagonal bases, which are defined by $|↗\;\rangle =\left(|↑\;\rangle +|↓\;\rangle \right)/\sqrt{2}$ and $|↘\;\rangle =\left(|↑\;\rangle -|↓\;\rangle \right)/\sqrt{2}$, respectively, for both spin and orbital angular momentum. The singlet state must be remained to be the singlet in diagonal and horizontal bases as $|\;\mathrm{Singlet}\;\rangle ={{{{(|↘\;\rangle }_{s}|↗\;\rangle }_{o}-|↗\;\rangle }_{s}|↘\;\rangle }_{o})/\sqrt{2}$, since the total spin after the coupling must vanish. The singlet state corresponds to the point at $\langle U_{1}\rangle =-1$ (Fig. 1 (B)) on the higher-order Poincaré sphere. On the other hand, the triplet state becomes $|\;\mathrm{Triplet}\;\rangle ={{{{(|↗\;\rangle }_{s}|↗\;\rangle }_{o}-|↘\;\rangle }_{s}|↘\;\rangle }_{o})/\sqrt{2}$. The triplet state corresponds to the point at $\langle U_{1}\rangle =1$ (Fig. 1 (B)) on the sphere. These states are distinguishable from images, since diagonal and anti-diagonal states correspond to images of dipoles, which are pointing diagonal and anti-diagonal directions, respectively.

Thus, in order to confirm singlet and triplet states, we have inserted PL2 in front of the NPBS (Fig. 2 (b)). This corresponds to the Bell projection for classically entangled coherent photons [23], [26], [27], [28], [15], [29], [30]. PL2 was rotated to change the direction of the projection, which was monitored by the PM. The polarisation state at the CMOS camera is mirror-reflected by the NPBS, such that the diagonal state at the PM should be considered for the anti-diagonal state at the CMOS camera, and *vice versa*.

**Figure 11:**
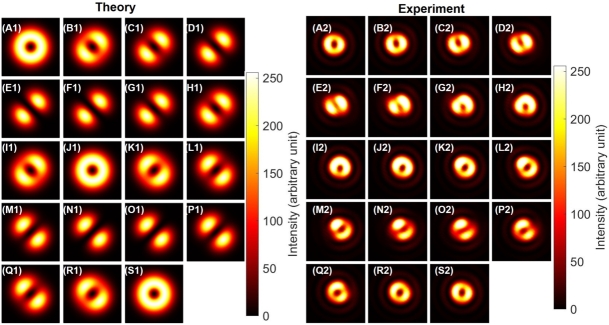
Photonic singlet state, realised by the superposition among orthogonal states for both spin and orbital angular momentum. (A1)-(S1) theoretical calculations. (A2)-(S2) Experimental data. Polariser was set to be rotated from (A) horizontal at 0^∘^, (J) vertical at 90^∘^, and back to (S) horizontal at 180^∘^ with the step of 10^∘^. Far-field images are shown for (A) and (S) a pure right twisted state with the horizontal polarisation, and (J) a pure left twisted state with the vertical polarisation. For the diagonal polarisation at (N)-(O), and the anti-diagonal polarisation at (E)-(F), dipoles are pointing along orthogonal directions to the polarisation, respectively, showing total angular momentum of zero.

The experimental images are shown in Figs. 11 (A2)-(S2) for the singlet state, together with theoretical calculations (Figs. 11 (A1)-(S1)) only for intensity profiles, since the phase is projected by a rotated polariser. Here, we have adjusted the phase to be singlet, by setting the polariser to be aligned for the diagonal direction at the CMOS camera, and then, HWP4 was rotated to confirm the dipole is aligned to be anti-diagonal direction, as shown in Fig. 11 (N2). After setting the phase to be singlet, we have observed the images after projecting the polarisation state by PL2.

Among these images, Fig. 11 (A2) corresponds to the horizontally polarised state by the projection from the singlet to ${{|↑\;\rangle }_{s}|↓\;\rangle }_{o}$, such that the topological charge must be -1 for the right vortex. On the other hand, Fig. 11 (J2) corresponds to the vertically polarised state by the projection to ${{-|↓\;\rangle }_{s}|↑\;\rangle }_{o}$, such that the topological charge must be one for the left vortex. Unfortunately, we cannot distinguish the chirality of the vortices nor the phase to determine whether the state is singlet or triplet.

Nevertheless, by rotating PL2, we confirmed expected dipole images. When we set the PL2 to the diagonal direction, the polarisation state at the CMOS (Complementary Metal-Oxide-Semiconductor) camera must be anti-diagonal due to one mirror reflection at the NPBS. The image corresponding to this situation is shown in Fig. 11 (E2), where the dipole is pointing to the diagonal direction. This was exactly what we expected for the singlet state, since we expected projection to ${{|↘\;\rangle }_{s}|↗\;\rangle }_{o}$. Similarly, if we align PL2 to the anti-diagonal direction, the polarisation state at the CMOS camera must be diagonal, and we expected the projection to ${{-|↗\;\rangle }_{s}|↘\;\rangle }_{o}$, which was confirmed in Fig. 11 (N2), whose dipole is along the anti-diagonal direction.

**Figure 12:**
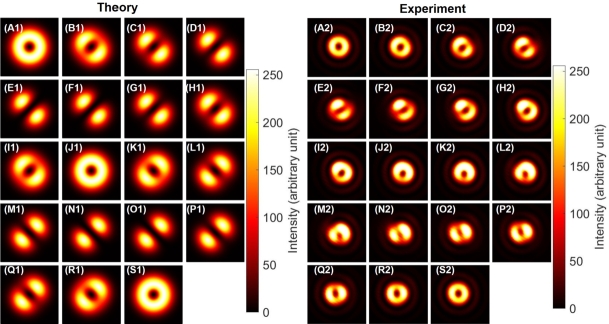
Photonic triplet state, realised by the superposition among orthogonal states for both spin and orbital angular momentum. (A1)-(S1) theoretical calculations. (A2)-(S2) Experimental data. Polariser was set to be rotated from (A) horizontal at 0^∘^, (J) vertical at 90^∘^, and back to (S) horizontal at 180^∘^ with the step of 10^∘^. Dipoles are aligned to the same diagonal and anti-diagonal directions with the directions of polarisation.

We have also examined the triplet state. First, we have set the phase, similar to the singlet state. After aligning PL2 to the anti-diagonal direction for the polarisation at the CMOS camera, we have rotated HPW4 to align the dipole to the same diagonal direction, seen in Fig. 12 (N2). Then, we have taken images after projecting the polarisation state to the direction defined by PL2, and confirmed that the dipoles are aligned to the same direction with the polarisation, as expected from the simple calculations for SU(4) states, shown above.

We have conducted the physical rotations of PL2, exactly the same way both for the singlet (Fig. 11) and the triplet (Figs. 12 (A1)-(S1) and Figs. 12 (A2)-(S2)). states, however, the rotations in the Poincaré sphere for orbital angular momentum are opposite each other (Fig. 1). For the singlet state, the states after the projection were changed from the right-twisted state, the diagonal dipole state, the left-twisted state, and to the anti-diagonal dipole state. Contrary, for the triplet state, the states after the projection were changed from the right-twisted state, the anti-diagonal dipole state, the left-twisted state, and to the diagonal dipole state. This difference for the direction of the rotation on the Poincaré sphere for orbital angular momentum is simply explained by the SU(4) states, given by the superposition states of two SU(2) states for spin and orbital angular momentum (Visualization 2).

**Figure 13:**
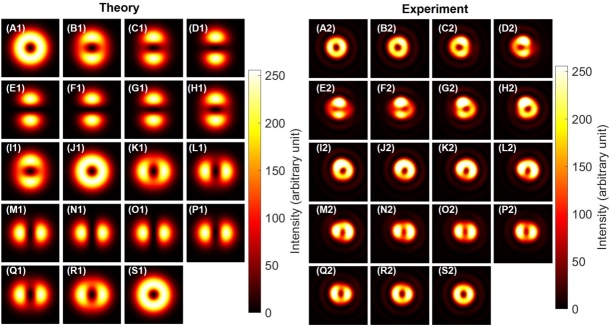
Rotated singlet state of spin and orbital angular momentum. (A1)-(S1) theoretical calculations. (A2)-(S2) Experimental data. Polariser was set to rotated to be (A) horizontal at 0^∘^, (J) vertical at 90^∘^, and back to (S) horizontal at 180^∘^ with the step of 10^∘^. Vertical dipoles are realised under the anti-diagonal polarisation states, and horizontal dipoles are realised under the diagonal polarisation states.

**Figure 14:**
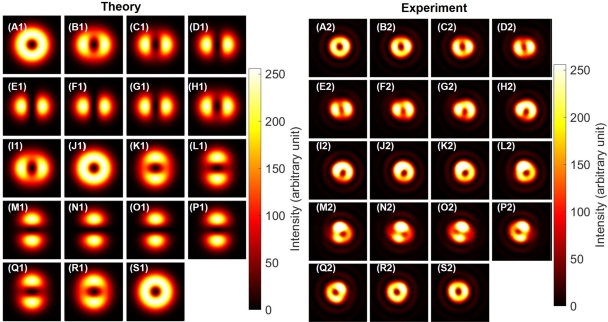
Rotated triplet state of spin and orbital angular momentum. (A1)-(S1) theoretical calculations. (A2)-(S2) Experimental data. Polariser was set to rotated to be (A) horizontal at 0^∘^, (J) vertical at 90^∘^, and back to (S) horizontal at 180^∘^ with the step of 10^∘^. Horizontal dipoles are realised under the anti-diagonal polarisation states, and vertical dipoles are realised under the diagonal polarisation states.

We also examined the projection from SU(4) states to SU(2)×SU(2) by the polariser for states with horizontal and vertical dipoles, as shown in Figs. 13 (A1)-(S1), Figs. 13 (A2)-(S2), Figs. 14 (A1)-(S1), and Figs. 14 (A2)-(S2), by further phase-shifting the singlet and the triplet states, respectively. For the experiments, shown in Fig. 13, we have adjusted the phase to align the dipole to the horizontal direction under the diagonally polarisation state (Figs. 13 (N)-(O)). Then, the rotated singlet state is projected to show the vertical dipole under the anti-diagonally polarisation state (Figs. 13 (E)-(F)).

Similarly, for the experiments in Fig. 14, we have adjusted the phase to align the dipole to the vertical direction under the diagonally polarisation state (Figs. 14 (N)-(O)). Then, the rotated triplet state is projected to show the horizontal dipole under the anti-diagonally polarisation state (Figs. 14 (E)-(F)). Therefore, the direction of rotations becomes opposite between rotated singlet and triplet states in the Poincaré sphere for the orbital angular momentum.

All these experiments are consistent with a simple quantum theory of SU(4) states. After the projection to chose one polarisation state, one of the polarisation state is chosen, and the orbital angular momentum state is also projected by the selection. The ray after passing through the polariser is described by the SU(2)×SU(2) state, since the ray has certain polarisation state with certain orbital angular momentum state. The ray in the SU(2)×SU(2) can be still manipulated by wave-plates or other optical components, before observing the states at the CMOS camera. But, the states of the ray cannot cover the full SU(4) states, spanned by four orthogonal states, since they are not described by the superposition state of orthogonal basis states of SU(4), any more. In order to recover the full symmetry of SU(4), we need the set up similar to the whole experiment, shown in Fig. 2 (b), to allow the splitting of the ray to recombine after rotational operations for spin and orbital angular momentum. Mathematically, operators in SU(2)×SU(2) are block diagonalised to conduct separate rotations for spin and orbital angular momentum, individually, while the SU(4) operations are necessary to realise singlet and triplet states. Our experiments may serve as a platform to explore the projection from SU(4) to SU(2)×SU(2). If we include the non-twisted state, we can realise a topological colour-charged states to realise SU(3) states for fixed polarisation. If we include two orthogonal polarisation states, we can prepare 6 orthogonal states to realise SU(6). In this case, we can explore the projection from SU(6) to SU(2)×SU(3). SU(6) is employed for a unified theory in elementary particle physics, while our experiments would be completely different from the mechanism related to the spontaneous symmetry breaking of the vacuum due to a phase-transition [69], [70], [71], [72], [73], [74], [75], [76], [110], [111], [112], [113], [114], [115], [116]. Nevertheless, it might provide some insights to understand the symmetry breaking and associated states, since our projection scheme is experimentally accessible and straightforward to observe the projected states as images.

## 5 Conclusion

We developed an SU(*N*) theory for coherent photons and found expectation values for generalised angular momentum are transferred in SO($N^{2}-1$) space. We also showed that the radius of the hypersphere for expectation values is conserved upon the unitary transformation and the value becomes $\sqrt{2(N-1)/N}$. It was also shown that expectation values are shown on N(N−1)/2 higher-order Poincaré spheres instead of the hypersphere [1], [2], [3], [4], [5], [6], [7], [8], [9], [10], [11], [12], [13], [14], [15], [16], [17], [18], [19], [20], [21], [22]. We confirmed that spin and orbital angular momentum of coherent photons are described by a theory of Lie group [87], [88], [117], [118], [119], [113], [96], [89], [90], [91], [92], [133], [93], [94], [60], [61], [62], [63], [64], [78], [79], [80], [81], [82], [83], [84], [85], [48]. The expectation values of spin angular momentum for the coherent photons are described by Stokes parameters [78], [81].

To examine the validity of the theory, we developed a simple experimental technique to control the polarisation states by the combination of quarter-waveplates and half-waveplates over the full Poincaré sphere. We also demonstrated to control the expectation value of orbital angular momentum by using waveplates and vortex lenses. By extending this technique, we also demonstrated to realise a superposition state between a twisted state and a fundamental Gaussian mode, and we observed the continuous motion of the topological charge upon changing the amplitude and the phase. This allows us to prepare SU(3) states, which can be assigned to topological colour-charge for realising photonic QCD [48]. By combining spin and orbital angular momentum, we could construct both singlet and triplet states, which were confirmed by the projection of the spin state by the polariser, whose images were consistent with theoretical expectations for SU(4) states. Our results show that we can realise SU(*N*) states by using spin and orbital angular momentum as far as *N* is reasonably small enough to be accessible by vortex lenses and waveplates.

## CRediT authorship contribution statement

**Shinichi Saito:** Writing – review & editing, Writing – original draft, Visualization, Validation, Supervision, Software, Resources, Project administration, Methodology, Investigation, Funding acquisition, Formal analysis, Data curation, Conceptualization.

## Declaration of Competing Interest

The authors declare the following financial interests/personal relationships which may be considered as potential competing interests: Shinichi Saito reports financial support was provided by 10.13039/501100001691Japan Society for the Promotion of Science. Shinichi Saito reports financial support was provided by Hitachi Ltd. If there are other authors, they declare that they have no known competing financial interests or personal relationships that could have appeared to influence the work reported in this paper.

## Data Availability

The data that support the findings of this study is available within the article. The raw data supporting the conclusion of this article will be made available by the author upon reasonable requests.

## Appendix A Supplementary material

Video 1 Superposition states between a left-twisted state and a no-vortex state under vertical polarisation. Topological charge moves upon changing the amplitudes by rotating the half waveplate. Video 1

Video 2 Photonic singlet (left) and triplet (right) states observed after passing through a rotated polariser. Video 2
